# Ad4BP/SF-1 regulates cholesterol synthesis to boost the production of steroids

**DOI:** 10.1038/s42003-018-0020-z

**Published:** 2018-03-22

**Authors:** Takashi Baba, Hiroyuki Otake, Miki Inoue, Tetsuya Sato, Yasuhiro Ishihara, Ju-Yeon Moon, Megumi Tsuchiya, Kanako Miyabayashi, Hidesato Ogawa, Yuichi Shima, Lixiang Wang, Ryuichiro Sato, Takeshi Yamazaki, Mikita Suyama, Masatoshi Nomura, Man Ho Choi, Yasuyuki Ohkawa, Ken-ichirou Morohashi

**Affiliations:** 1 Department of Molecular Biology, Graduate School of Medical Sciences, Kyushu University, Maidashi 3-1-1, Higashi-ku, Fukuoka, 812-8582 Japan; 2 Department of Systems Life Sciences, Graduate School of Systems Life Sciences, Kyushu University, Maidashi 3-1-1, Higashi-ku, Fukuoka, 812-8582 Japan; 3 Division of Bioinformatics, Medical Institute of Bioregulation, Kyushu University, Maidashi 3-1-1, Higashi-ku, Fukuoka, 812-8582 Japan; 4 Laboratory of Molecular Brain Science, Graduate School of Integrated Arts and Sciences, Hiroshima University, Kagamiyama 1-7-1, Higashi-Hiroshima, 739-8521 Japan; 5 Molecular Recognition Research Center, Korea Institute of Science and Technology, Seoul, 02792 Korea; 6 Nuclear Dynamics Group, Graduate School of Frontier Biosciences, Osaka University, Yamadaoka 1-3, Osaka, 565-0871 Japan; 7 Department of Anatomy, Kawasaki Medical School, 577 Matsushima, Kurashiki, 701-0192 Japan; 8 Department of Medicine and Bioregulatory Science, Graduate School of Medical Sciences, Kyushu University, Maidashi 3-1-1, Higashi-ku, Fukuoka, 812-8582 Japan; 9 Department of Applied Biological Chemistry, Graduate School of Agricultural and Life Sciences, The University of Tokyo, 1-1-1 Yayoi, Bunkyo, Tokyo, 113-8657 Japan; 10 Division of Endocrinology and Metabolism, Department of Internal Medicine, Kurume University School of Medicine, Asahimachi 67, Kurume, 830-0011 Japan; 11 Division of Transcritomics, Medical Institute of Bioregulation, Kyushu University, Maidashi 3-1-1, Higashi-ku, Fukuoka, 812-8582 Japan

## Abstract

Housekeeping metabolic pathways such as glycolysis are active in all cell types. In addition, many types of cells are equipped with cell-specific metabolic pathways. To properly perform their functions, housekeeping and cell-specific metabolic pathways must function cooperatively. However, the regulatory mechanisms that couple metabolic pathways remain largely unknown. Recently, we showed that the steroidogenic cell-specific nuclear receptor Ad4BP/SF-1, which regulates steroidogenic genes, also regulates housekeeping glycolytic genes. Here, we identify cholesterogenic genes as the targets of Ad4BP/SF-1. Further, we reveal that Ad4BP/SF-1 regulates *Hummr*, a candidate mediator of cholesterol transport from endoplasmic reticula to mitochondria. Given that cholesterol is the starting material for steroidogenesis and is synthesized from acetyl-CoA, which partly originates from glucose, our results suggest that multiple biological processes involved in synthesizing steroid hormones are governed by Ad4BP/SF-1. To our knowledge, this study provides the first example where housekeeping and cell-specific metabolism are coordinated at the transcriptional level.

## Abstract (web summary)

Takashi Baba and colleagues show that the steroidogenic cell-specific nuclear receptor Ad4BP/SF-1 targets cholesterogenic genes. Given that cholesterol is a precursor of the steroid hormones, this study suggests that Ad4BP/SF-1 regulates the synthesis of cholesterol to boost the production of steroids.

## Introduction

Steroid hormones vital for diverse biological processes are synthesized from cholesterol through multi-step reactions^1–4^. Steroidogenesis is active only in functionally specialized steroidogenic cells, such as adrenocortical and testicular Leydig cells. Multiple studies have established that all steroidogenic genes are regulated by the steroidogenic cell-specific transcription factor Ad4BP/SF-1 (NR5A1), which binds them directly^5–8^. Recently, we showed that this transcription factor also directly regulates nearly all glycolytic genes, and thus also governs the expression of genes involved in housekeeping metabolism^9^. At that time, however, we could not determine why this steroidogenic cell-specific transcription factor regulates two apparently independent metabolic pathways, steroidogenesis and glycolysis.

Because cholesterol is an essential component of all cells, its cellular levels are maintained within a narrow range by a complex control system that balances incorporation, excretion, de novo synthesis, and consumption^10–12^. This control machinery is unique in that sensing of intracellular cholesterol concentration regulates proteolytic cleavage of the membrane-bound inactive form of a transcription factor, SREBP-2 (sterol-responsive element binding protein-2), into its active soluble form. Following proteolysis, the soluble form of SREBP-2 translocates into the nucleus to directly activate many cholesterogenic genes^11^.

In addition to its role as a key component of cellular membranes, cholesterol is used as the starting material for steroidogenesis in steroidogenic cells^1–3^. Therefore, it is reasonable that steroidogenic cells would have a unique mechanism for meeting their augmented need for cholesterol. Indeed, it was reported that steroidogenic cells primarily use intracellular cholesterol stores for steroidogenesis in response to steroidogenic stimulation. Then, after the cholesterol stores decreased rapidly, de novo cholesterogenesis is activated and the synthesized cholesterol is used for steroidogenesis^13^. As mentioned above, Ad4BP/SF-1 regulates the genes implicated in both glycolytic and steroidogenic pathways. Given that pyruvate, the final product of glycolysis, can be converted to acetyl-CoA, the starting material for cholesterogenesis, we hypothesize that the glycolytic and steroidogenic pathways might be connected via the cholesterogenic pathway. Given the interconnections among these three metabolic pathways, we predict that Ad4BP/SF-1 would also regulate cholesterogenic genes, thereby collectively governing all metabolic processes from the one that utilizes glucose to the one that produces steroid hormones (Supplementary Fig. 1).

## Results

### Indirect regulation of cholesterogenic genes by Ad4BP/SF-1

**Fig. 1:**
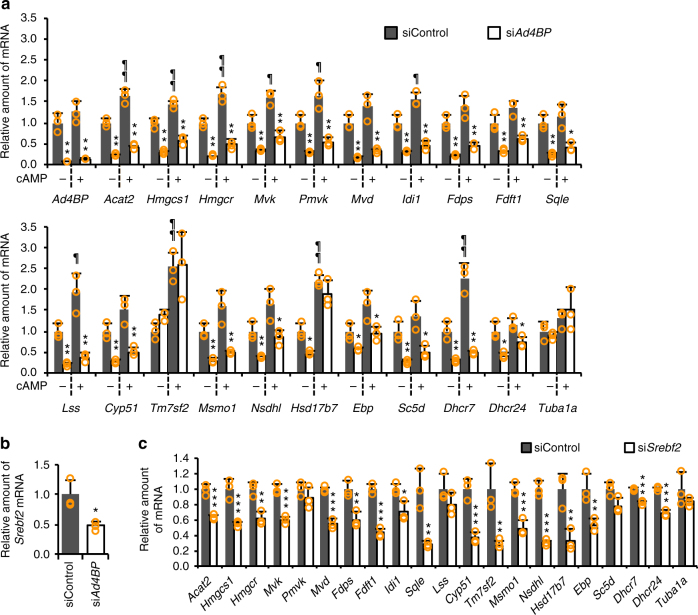
Cholesterogenic gene expression affected by *Ad4BP/SF-1* knockdown. **a** Cholesterogenic gene expression in *Ad4BP/SF-1* knockdown or control Y-1 cells in the absence or presence of cAMP was determined by qRT-PCR. Bars with “*” are compared to siControl-treated cells. *p < 0.05, **p < 0.01. Bars with an “^¶^” are compared to vehicle-treated cells; ^¶^*p* < 0.05, ^¶¶^*p* < 0.01. **b** *Srebf2* expression was determined in *Ad4BP/SF-1* knockdown or control Y-1 cells by qRT-PCR. *p < 0.01. **c** Cholesterogenic gene expression was determined in *Srebf2* knockdown or control Y-1 cells by qRT-PCR. *p < 0.05, **p < 0.025, ***p < 0.005. **a**–**c** Average values with SDs (*n* = 3) are indicated. *Tuba1a* was used as a negative control. The values for the siControl-treated cells were normalized to 1.0

To explore the possibility that Ad4BP/SF-1 is involved in cholesterogenic gene regulation, we obtained the transcriptomes of Y-1 cells, which are derived from a mouse adrenocortical tumor. The mRNA-seq data revealed that expression of 16 out of 20 cholesterogenic genes decreased below 70% of control levels in *Ad4BP/SF-1* knockdown cells (Supplementary Fig. 2a). Consistent with the transcriptome data, quantitative RT-PCR (qRT-PCR) confirmed that the cholesterogenic genes, with the exception of *Tm7sf2*, were down-regulated by the knockdown (Fig. 1a, Supplementary Fig. 2b). Cyclic AMP (cAMP) stimulates steroidogenic gene expression^4^. Hence, we investigated whether cholesterogenic gene expression is activated by cAMP. Indeed, we found that expression of many cholesterogenic genes was increased by cAMP stimulation (Fig. 1a). In *Ad4BP/SF-1* knockdown cells, the cAMP-induced up-regulation of cholesterogenic genes (except for *Tm7sf2* and *Hsd17b7*) was reduced (Fig. 1a).

We next investigated whether similar knockdown effects would be observed in steroidogenic Leydig cells prepared from mouse testes. When these cells were cultured in the absence of cAMP, transcriptome analysis and qRT-PCR revealed that cholesterogenic genes were not collectively down-regulated by *Ad4BP/SF-1* knockdown (Supplementary Fig. 3a and b). However, when the expression of these genes was activated by cAMP, they were mostly down-regulated by the knockdown (Supplementary Fig. 3a and b). Together, these findings suggest that Ad4BP/SF-1 is implicated in transcriptional regulation of nearly all cholesterogenic genes in adrenocortical Y-1 and cAMP-stimulated testicular Leydig cells.

SREBP-2, encoded by *Srebf2*, is a key regulator of cholesterogenic gene transcription^11^. Because a chromatin immunoprecipitation-sequencing (ChIP-seq) data set for SREBP-2 obtained from hepatocyte was available^14^, we investigated whether SREBP-2 ChIP peaks are present in cholesterogenic genes. Indeed, SREBP-2 ChIP peaks were detected in all cholesterogenic genes (Supplementary Fig. 4). Furthermore, *Srebf2* was down-regulated by *Ad4BP/SF-1* knockdown (Fig. 1b, Supplementary Fig. 5a and b). Cholesterogenic genes are up-regulated by SREBP-2 activated in response to a lowered intracellular cholesterol level^10–12^. Thus, we expected that the decreased expression of *Srebf2* leads to a reduction in active SREBP-2 protein, irrespective of intracellular cholesterol contents, and as a consequence, cholesterogenic genes are down-regulated. Indeed, *Srebf2* knockdown (Supplementary Fig. 5c) resulted in down-regulation of many (17 out of 20) cholesterogenic genes in Y-1 cells (Fig. 1c, Supplementary Fig. 5d). These results raise the possibility that Ad4BP/SF-1 controls cholesterogenic genes indirectly by regulating *Srebf2* transcription.

### Direct regulation of cholesterogenic genes by Ad4BP/SF-1

**Fig. 2:**
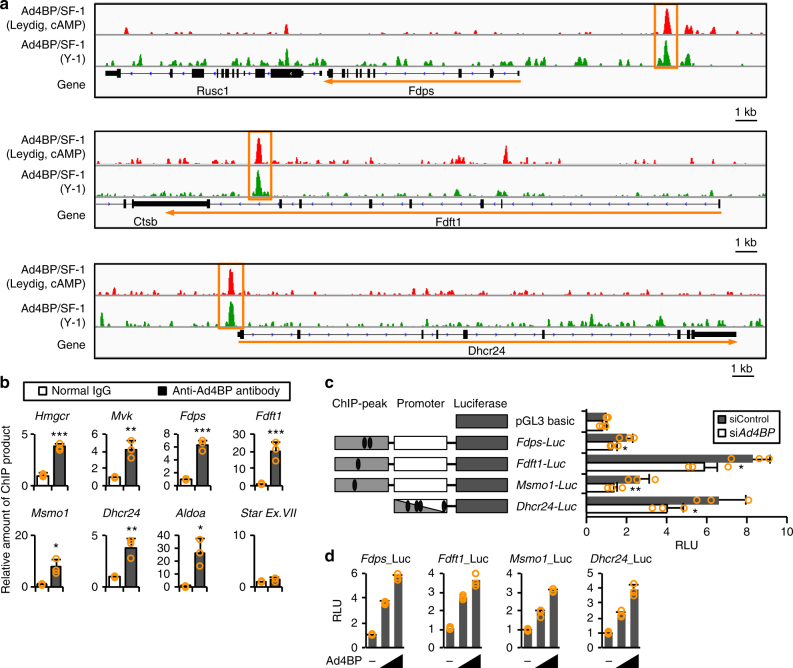
Regulation of cholesterogenic genes by Ad4BP/SF-1. **a** Ad4BP/SF-1 ChIP peaks in Y-1 and cAMP-treated Leydig cells are shown for the indicated cholesterogenic genes (*Fdps*, *Fdft1*, and *Dhcr24*). The ChIP peaks are enclosed by orange squares. Peaks for other cholesterogenic genes are shown in Supplementary Fig. 4. **b** Accumulation of Ad4BP/SF-1 on cholesterogenic genes was confirmed by ChIP-qPCR. *Star* Ex.VII was used as a negative control. *p < 0.05, **p < 0.01, and ***p < 0.005. *n* = 3. **c** Luciferase reporter gene constructs, *Fdps*-Luc, *Fdft1*-Luc, *Msmo1*-Luc, and *Dhcr24*-Luc, are shown (left). Gray squares represent genome fragments where accumulation of Ad4BP/SF-1 was observed by the ChIP-seq. White squares represent genome fragments corresponding to promoters of the genes. For *Dhcr24*, accumulation of Ad4BP/SF-1 was detected in the promoter region. Ovals represent potential Ad4BP/SF-1-binding sites. The reporters were transfected into Y-1 cells with si*Ad4BP/SF-1* or siControl. Average RLU (relative luciferase unit) values and SDs of the luciferase activities are indicated. The average value for pGL3 basic in the siControl-treated cells was normalized to 1.0. *p < 0.05 and **p < 0.01. *n* = 3. **d** The reporters were transfected into HeLa cells with increasing amounts of the expression vector for Ad4BP/SF-1. Average values for pGL3 basic in the absence of the Ad4BP/SF-1 expression vector were normalized to 1.0

In addition to the indirect regulation described above, it still remains the possibility that Ad4BP/SF-1 directly regulates cholesterogenic genes. To explore this possibility, we examined previously published ChIP-seq data sets obtained from Y-1 cells^9^ to ask whether Ad4BP/SF-1 binds directly to cholesterogenic genes. In addition, we obtained new Ad4BP/SF-1 ChIP-seq data sets from cAMP-treated or cAMP-untreated Leydig cells. We found that Ad4BP/SF-1 bound to many cholesterogenic genes (11 out of 20 in Y-1, and 12 out of 20 in cAMP-treated and cAMP-untreated Leydig cells) (Fig. 2a, Supplementary Fig. 4, Supplementary Table 1). Accumulation of Ad4BP/SF-1 was confirmed by chromatin immunoprecipitation-quantitative PCR (ChIP-qPCR) (Fig. 2b). As expected, all ChIP-peak regions contained single or multiple consensus binding sequences of Ad4BP/SF-1, and at least one binding sequence was conserved in the corresponding regions of the human genes (Supplementary Table 1). We next investigated whether the ChIP-peak regions are associated with transcriptional activity, using luciferase reporter genes containing the ChIP-peak regions and promoters of *Fdps*, *Fdft1*, *Msmo1*, and *Dhcr24*. These regions exhibited transcriptional activity in Y-1 cells, and their activity was suppressed by *Ad4BP/SF-1* knockdown (Fig. 2c). Consistent with this, the reporter genes were activated in non-steroidogenic HeLa cells by ectopically expressed Ad4BP/SF-1 in a dose-dependent manner, suggesting that a number of cholesterogenic genes are directly regulated by Ad4BP/SF-1 (Fig. 2d).

### Cooperative regulation by Ad4BP/SF-1 and SREBP-2

**Fig. 3:**
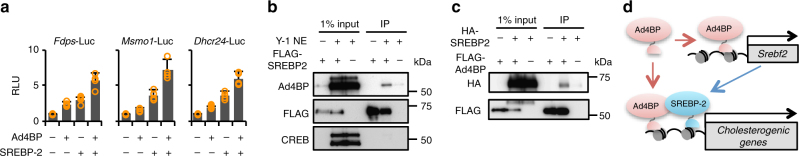
Cooperative activation of cholesterogenic genes by Ad4BP/SF-1 and SREBP-2 possibly through mutual interaction. **a** Luciferase reporter gene constructs, *Fdps*-Luc, *Msmo1*-Luc, and *Dhcr24*-Luc, were transfected into Y-1 cells with expression vectors for Ad4BP/SF-1 and SREBP-2. Average values and SDs of the luciferase activities are indicated. Average values in the absence of the expression vectors were normalized to 1.0. *n* = 4. **b** Purified FLAG-SREBP-2 protein was mixed with nuclear extracts prepared from Y-1 cells, and then proteins interacting with SREBP-2 were immunoprecipitated with anti-FLAG antibody. The immunoprecipitates were subjected to western blotting using antibodies for Ad4BP/SF-1, FLAG, and CREB. CREB was used as a negative control. Full blot images are shown in Supplementary Fig. 10. NE nuclear extract. **c** Purified HA-SREBP-2 and FLAG-Ad4BP/SF-1 were mixed, and then immunoprecipitated with anti-FLAG antibody. The immunoprecipitate was subjected to western blotting with anti-HA antibody to examine whether HA-SREBP-2 was co-immunoprecipitated. **d** A model of cholesterogenic genes regulation is illustrated. Ad4BP/SF-1 possibly regulates the cholesterogenic genes by two ways. Ad4BP/SF-1 directly binds to cholesterogenic genes and then regulates the genes possibly in cooperation with SREBP-2. Besides, Ad4BP/SF-1 indirectly regulates cholesterogenic genes through *Srebf2* gene regulation

The locations of the ChIP peaks of SREBP-2 and Ad4BP/SF-1 exhibited distinct features: the former accumulated near the transcription initiation sites (putative promoter regions) of all cholesterogenic genes (Supplementary Fig. 4), whereas the latter tended to accumulate far from the promoters. Hence, we investigated the functional correlation between Ad4BP/SF-1 and SREBP-2. Similar to Ad4BP/SF-1, SREBP-2 alone activated the reporter gene transcription (Fig. 3a). Moreover, these two factors cooperatively activated the reporter genes (Fig. 3a). In regard to the mechanism underlying the cooperative action of Ad4BP/SF-1 and SREBP-2, we hypothesized that the two transcription factors physically interact. When purified FLAG-tagged SREBP-2 was incubated with Y-1 cell nuclear extract, and the mixture was subjected to immunoprecipitation with an anti-FLAG antibody, the immunoprecipitates contained Ad4BP/SF-1 (Fig. 3b). Moreover, direct protein–protein interaction was confirmed by an in vitro binding experiment using purified FLAG-Ad4BP/SF-1 and HA-SREBP-2 (Fig. 3c). Together, these findings indicate that Ad4BP/SF-1 regulates nearly all cholesterogenic genes in two ways: direct regulation in cooperation with SREBP-2, and indirect regulation through transcriptional induction of the *Srebf2* gene (Fig. 3d).

### Cholesterogenesis reduced by *Ad4BP/SF-1* knockdown

**Fig. 4:**
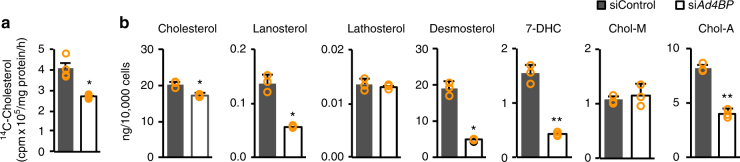
Cholesterogenesis affected by *Ad4BP/SF-1* knockdown. **a** The activity of de novo cholesterogenesis was determined by quantification of ^14^C-cholesterol synthesized from ^14^C-acetate. The amounts of ^14^C-cholesterol in *Ad4BP/SF-1* knockdown and control Y-1 cells are shown. Average values and SDs are indicated. *p < 0.01. *n* = 4. **b** The amounts of cholesterol, intermediate products (lanosterol, lathosterol, desmosterol, and 7-DHC), and esterified cholesterols (Chol-M and Chol-A) were determined in *Ad4BP/SF-1* knockdown and control Y-1 cells. Average values and SDs are indicated. *p < 0.05, **p < 0.01. *n* = 3. 7-DHC 7-dehydrocholesterol, Chol-M cholesteryl myristate, Chol-A cholesteryl arachidonate. Average values and SDs are indicated

These observations raised the possibility that *Ad4BP/SF-1* knockdown decreases cholesterogenic activity. To measure activity, we cultured Y-1 cells in medium containing ^14^C-acetate, and then determined the amount of de novo synthesized ^14^C-cholesterol. As expected, the amount of ^14^C-cholesterol was decreased to 66% of control levels in *Ad4BP/SF-1* knockdown cells (Fig. 4a). Consistent with this, the amount of cellular cholesterol was mildly (~15%) decreased by *Ad4BP/SF-1* knockdown (Fig. 4b). The levels of the intermediate products lanosterol, desmosterol, and 7-dehydrocholesterol were decreased by the knockdown, whereas the level of lathosterol was unaffected. The level of cholesteryl-arachidonate, the major cholesteryl ester in the adrenal cortex^15^, also decreased, whereas the level of cholesteryl-myristate was not changed. Together, these findings indicate that *Ad4BP/SF-1* knockdown reduced cholesterol synthesis through the expression of cholesterogenic genes.

### Cholesterogenesis and steroidogenesis bridged by Ad4BP/SF-1

If the glycolytic and steroidogenic pathways are connected via the cholesterogenic pathway, Ad4BP/SF-1 may govern multiple metabolic pathways from glycolysis to steroidogenesis. To test this hypothesis, two gaps between the metabolic pathways remained to be bridged.

The first of these gaps is between cholesterogenesis and steroidogenesis. In steroidogenic cells, cholesterol is supplied via de novo cholesterogenesis and receptor-mediated endocytosis of low-density and high-density lipoproteins^13,16–18^. *Ldlr* and *Scarb1* encode the receptors responsible for the endocytosis of low-density and high-density lipoproteins, respectively. Similar to the cholesterogenic genes, mRNA-seq studies showed that the expression of these receptor genes was decreased by *Ad4BP/SF-1* knockdown, whereas that of genes related to excretion of cholesterol (*Abca1* and *Abcg1*)^19,20^ was not altered (Supplementary Fig. 6a). The endocytosed lipoproteins are hydrolyzed in lysosomes, and then cholesterol is transported to endoplasmic reticula^21^, where the final reaction of cholesterogenesis takes place. Because CYP11A1, which catalyzes the initial reaction of steroidogenesis, is localized in the mitochondrial inner membrane^22^, cholesterol must be transported from endoplasmic reticula to the mitochondrial inner membrane. This transport consists of two steps: first from endoplasmic reticula to mitochondrial outer membrane, and the second from the outer membrane to inner membrane^22^. The STAR protein mediates the transport of cholesterol from mitochondrial outer membrane to inner membrane, and the encoding gene is regulated by Ad4BP/SF-1^23^.

**Fig. 5:**
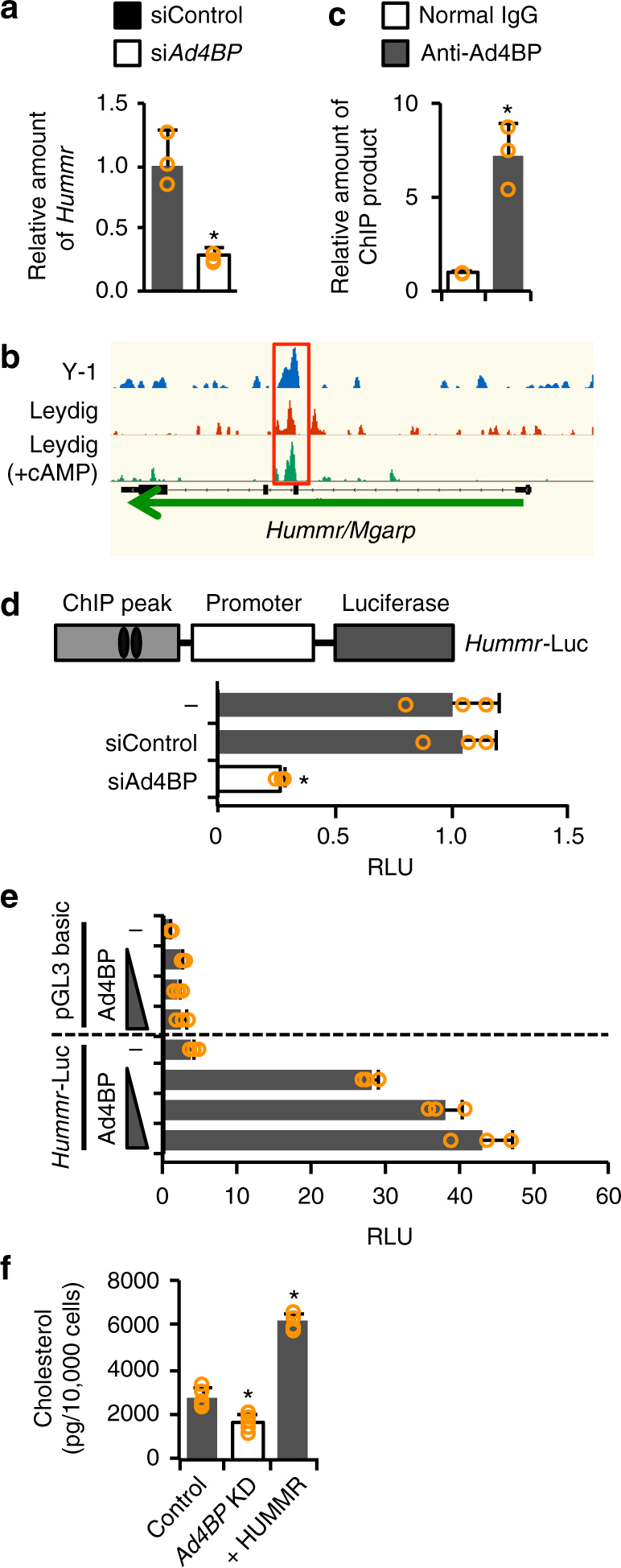
Regulation of *Hummr* by Ad4BP/SF-1. **a** The expression of *Hummr* in *Ad4BP/SF-1* knockdown or control Y-1 cells was examined by qRT-PCR. *p < 0.05. *n* = 3. **b** Ad4BP/SF-1 ChIP peaks at the *Hummr* gene in Y-1 and Leydig cells in the presence or absence of cAMP are shown. The ChIP peaks are enclosed by a red square. **c** Accumulation of Ad4BP/SF-1 to the ChIP peak of *Hummr* gene was confirmed by ChIP-qPCR. *p < 0.05. *n* = 3. **a**, **c** Average values and SDs are shown. The average value of the control was normalized to 1.0. **d** A luciferase reporter gene, *Hummr*-Luc, was constructed with the genome fragments where Ad4BP/SF-1 was accumulated (gray square) and the promoter of *Hummr* gene (white square). Ovals represent potential Ad4BP/SF-1-binding sites. The reporter was transfected into Y-1 cells with si*Ad4BP/SF-1* or siControl. Average values and SDs of the luciferase activities are indicated. The average value in the absence of siRNA was normalized to 1.0. *p < 0.05. *n* = 3. **e** Effects of Ad4BP/SF-1 overexpression on *Hummr*-Luc was examined. The reporter was transfected into HeLa cells with increasing amounts of the expression vector for Ad4BP/SF-1. Average values and SDs of luciferase activities are indicated. The average value for pGL3 basic in the absence of the expression vector was normalized to 1.0. *n* = 3. **f** Levels of mitochondrial cholesterol in control, *Ad4BP/SF-1* knockdown, and *Hummr*-overexpressing *Ad4BP/SF-1* knockdown Y-1 cells. Average values and SDs are shown. *p < 0.05. *n* = 5

Importantly, HUMMR (hypoxia up-regulated mitochondrial movement regulator, also called MGARP or OSAP) was identified recently as a plausible candidate for the mediator of cholesterol transport from endoplasmic reticula to mitochondrial outer membrane^24^. Therefore, we investigated whether Ad4BP/SF-1 regulates the *Hummr* gene. qRT-PCR and mRNA-seq revealed that *Hummr* expression was decreased by *Ad4BP/SF-1* knockdown in both Y-1 and Leydig cells, irrespective of cAMP treatment (Fig. 5a, Supplementary Fig. 6b, c). Furthermore, ChIP-seq revealed a clear Ad4BP/SF-1 peak at the *Hummr* locus in both Y-1 and Leydig cells (Fig. 5b). The accumulation of Ad4BP/SF-1 on the *Hummr* locus was confirmed by ChIP-qPCR (Fig. 5c). In addition, activity of a luciferase reporter gene carrying the ChIP-peak and promoter region of *Hummr* was suppressed by *Ad4BP/SF-1* knockdown (Fig. 5d, Supplementary Fig. 6d) and activated by ectopically expressed Ad4BP/SF-1 in a dose-dependent manner (Fig. 5e), indicating that the *Hummr* gene is regulated by Ad4BP/SF-1 via the ChIP-peak region.

Given that Ad4BP/SF-1 regulates *Hummr* gene, *Ad4BP/SF-1* knockdown should reduce the amount of mitochondrial cholesterol. Accordingly, we determined the amount of mitochondrial cholesterol in knockdown Y-1 cells, and found that it was indeed reduced to approximately 60% of control levels in the knockdown cells (Fig. 5f). Furthermore, overexpression of HUMMR in *Ad4BP/SF-1* knockdown cells markedly increased mitochondrial cholesterol (Fig. 5f), suggesting that the decrease was primarily due to reduced expression of *Hummr*. These results suggest that Ad4BP/SF-1 bridges the gap between the cholesterogenic and steroidogenic pathways by regulating *Hummr*, which has been implicated in cholesterol trafficking from endoplasmic reticula to mitochondria.

### Glycolysis and cholesterogenesis bridged by Ad4BP/SF-1

Next, we examined the involvement of Ad4BP/SF-1 in the regulation of the genes bridging the second gap between glycolysis and cholesterogenesis. The end product of glycolysis, pyruvate, is converted to cytosolic acetyl-CoA, which is a starting material for cholesterogenesis. At least nine genes encoding enzymes or transporters are involved in this conversion (Supplementary Fig. 7a). According to the transcriptome data, the expression of most, if not all, of these genes was affected by *Ad4BP/SF-1* knockdown, suggesting that many of them are the potential targets of Ad4BP/SF-1 (Supplementary Fig. 7b). Among them, ACLY (ATP citrate lyase) has been established as a key enzyme for the synthesis of cytosolic acetyl-CoA^25^, and its expression is regulated by SREBP-2^26^. Hence, we investigated *Acly* as a potential target of Ad4BP/SF-1 in steroidogenic cells. The ChIP-seq data demonstrated that Ad4BP/SF-1 accumulated in the *Acly* locus (Supplementary Fig. 8a), which was confirmed by ChIP-qPCR (Supplementary Fig. 8b). Consistent with this, *Acly* expression was decreased by *Ad4BP/SF-1* knockdown (Supplementary Fig. 8c). We then examined the transcription activity of the peak region using reporter gene assays. Reporter gene activity was suppressed by *Ad4BP/SF-1* knockdown (Supplementary Fig. 8d), and activated by overexpression of Ad4BP/SF-1 in a dose-dependent manner (Supplementary Fig. 8e). Together, these results suggest that *Acly* is a target gene of Ad4BP/SF-1. Given that ACLY is crucial for producing cytosolic acetyl-CoA, these results suggest that Ad4BP/SF-1 bridges the second gap from glycolysis to cholesterogenesis.

## Discussion

Cell-specific transcription factors have been studied in regard to their cell-specific functions, and thought to be specialized to regulate cell-specific genes. However, we previously reported that Ad4BP/SF-1 regulates housekeeping glycolytic genes, as well as cell-specific steroidogenic genes^9^. At that time, we could not explain why a tissue-specific transcription factor would be involved in housekeeping metabolic regulation. However, the results of this study suggest a novel mechanism by which this tissue-specific transcription factor integrates three distinct metabolic pathways—glycolysis, cholesterogenesis, and steroidogenesis—into a combined regulatory unit (Supplementary Fig. 1). ERRα (NR3B1) regulates genes involved in housekeeping energy metabolism, including glycolysis, the tricarboxylic acid (TCA) cycle, and oxidative phosphorylation^27–29^, whereas NRF2 regulates genes involved in the pentose phosphate pathway^30^. Considering their preferential expression in certain types of cells, these factors might also link housekeeping and cell-specific metabolism.

Given that Ad4BP/SF-1 is the predominant regulator of cholesterogenic genes in steroidogenic cells, we expected that expression of *Ad4BP/SF-1* and cholesterogenic genes would be synchronized under specific physiological conditions. We recently reported that during the differentiation of mouse fetal Leydig cells from their progenitors, the expression of *Ad4BP/SF-1* increases^31^. At the same time, the genes involved in glycolysis, the TCA cycle, oxidative phosphorylation, and steroidogenesis are activated^31^. Therefore, we investigated whether the genes newly identified as Ad4BP/SF-1 targets in this study are activated during differentiation. Similar to the metabolic genes above, the mRNA-seq data revealed that all cholesterogenic genes, as well as *Hummr* and *Acly*, were markedly up-regulated (Supplementary Fig. 9a). The up-regulation of cholesterogenic genes was confirmed by qRT-PCR (Supplementary Fig. 9b). Interestingly, *Srebf2* expression was only slightly increased, by less than 2-fold, whereas expression of cholesterogenic genes was increased by 2- to 22-fold (Supplementary Fig. 9a and b). Ad4BP/SF-1 itself was increased by 17-fold, suggesting that the elevated expression of cholesterogenic genes during differentiation of fetal Leydig cells is at least in part due to increased expression of Ad4BP/SF-1.

Although the liver is the principal organ involved in de novo synthesis of cholesterol from acetyl-CoA, most cell types, including adrenal cortex and testicular steroidogenic cells, have the ability to synthesize cholesterol^32,33^. The whole cholesterogenic pathway consists of multiple reactions executed by 20 enzymes. All genes encoding these enzymes have been regulated by SREBP-2^11^. Our investigation of the ChIP-seq data reported by Seo et al.^14^ revealed the accumulation of SREBP-2 near the transcription initiation sites (putative promoter regions) of all cholesterogenic genes, arguing in favor of the orchestrated regulation of all cholesterogenic genes by SREBP-2.

Our study implicates Ad4BP/SF-1, originally identified as the principal transcriptional regulator of steroidogenic genes, in control of cholesterogenic gene expression via regulation of *Srebf2* (encoding SREBP-2) and/or direct binding to the cholesterogenic genes themselves. Together, unlike hepatocytes, in which Ad4BP/SF-1 is not expressed, in steroidogenic cells cholesterogenic genes are regulated both by Ad4BP/SF-1 and SREBP-2. Because the demand for cholesterol is augmented in steroidogenic cells upon stimulation by pituitary hormones^4^, these cells may be uniquely equipped to meet this demand. Indeed, it was reported that *Scarb1*, which encodes high-density lipoprotein (HDL) receptor responsible for cholesterol incorporation, is directly regulated by Ad4BP/SF-1^18^. Our mRNA-seq result also suggested that low-density lipoprotein as well as HDL receptor genes, but not the genes required for cholesterol excretion (*Abca1* and *Abcg1*), are regulated by Ad4BP/SF-1. Taken together, by participating in the regulation of cholesterogenesis and perhaps cholesterol incorporation, Ad4BP/SF-1 provides an efficient mechanism for meeting the particular demand of cholesterol in the steroidogenic cells.

Steroidogenic cells do not store large amounts of steroids. Therefore, to secrete steroids upon stimulation, they must rapidly synthesize steroids by coordinating multiple routes that supply the materials for synthesis. Steroidogenic reactions consume NADPH, which is synthesized by multiple enzymes. Among them, malic enzyme and methylenetetrahydrofolate dehydrogenase make a significant contribution to NADPH synthesis^34,35^. Our recent study demonstrated that the genes encoding the enzymes mentioned above are direct target genes of Ad4BP/SF-1^36^. In addition, our previous study implicated Ad4BP/SF-1 in glycolytic gene regulation^9^. Glycolysis produces ATP, which is required for cholesterogenesis and for synthesis of cytosolic acetyl-CoA, mediated by ATP citrate lyase. Therefore, it is likely that glycolysis and cholesterogenesis are coupled by Ad4BP/SF-1 to balance the supply and the demand for ATP. Steroidogenesis is the most critical and specific function of steroidogenic cells. Together with our previously published results, the findings of this study suggest that Ad4BP/SF-1 orchestrates distinct metabolic pathways to supply the building blocks for steroidogenesis so that steroidogenic cells can accomplish their particular function (Supplementary Fig. 1).

In light of the possibility that Ad4BP/SF-1 might regulate glycolysis, cholesterogenesis, and steroidogenesis, as a unit, we noticed two gaps between these metabolic pathways. The first gap is between cholesterol trafficking from the endoplasmic reticula, where cholesterogenesis terminates, to the mitochondria, where steroidogenesis starts. Although not all components of the machinery have been identified, a recent study found that a principal component, HUMMR (also referred to as MGARP or OSAP), is critical for cholesterol transfer to the mitochondrial outer membrane^24^. Here, we showed that *Hummr* is regulated by Ad4BP/SF-1, suggesting that Ad4BP/SF-1 bridges the gap between cholesterogenesis and steroidogenesis.

As for the second gap, between glycolysis and cholesterogenesis, we identified *Acly* as a target gene of Ad4BP/SF-1. Acetyl-CoA synthesized by ACLY can be used for lipogenesis, as well as cholesterogenesis. Therefore, *Acly* may act downstream of both lipogenic and cholesterogenic regulation. Indeed, SREBP-1a and SREBP-2, which predominantly regulate the genes responsible for lipogenesis and cholesterogenesis, respectively, regulate *Acly* by binding to an SREBP-binding sequence in the promoter^26^. Therefore, Ad4BP/SF-1 and SREBP-2 together could bridge the gap between glycolysis and cholesterogenesis.

To our knowledge, our study provides the first example, where a single transcription factor can orchestrate housekeeping and cell-specific metabolism, enabling cells to effectively perform their particular functions.

## Methods

### Cell culture

Y-1 cells derived from a mouse adrenocortical tumor were grown in Dulbecco’s modified Eagle's medium (DMEM) (Wako, Tokyo, Japan) supplemented with 10% fetal bovine serum (FBS) and 1× penicillin-streptomycin (Invitrogen, Carlsbad, CA, USA) on collagen type I coated dishes (Iwaki, Tokyo, Japan). Mouse testicular Leydig cells were cultured in DMEM supplemented with 10% FBS and 1× penicillin–streptomycin on collagen type I coated dishes. HeLa cells derived from human cervical adenocarcinoma were grown in DMEM supplemented with 10% FBS and 1× penicillin–streptomycin.

### Isolation of Leydig cells

Leydig cells were isolated from mouse testes by Percoll density gradient centrifugation as previously described^37^. Briefly, testes harvested from ten 9-week-old male ICR mice were decapsulated and incubated for 10 min at 34 °C with gentle stirring with DMEM supplemented with 0.1% bovine serum albumin (BSA) (Roche, Basel, Swiss), 100 U/ml penicillin, 100 μg/ul streptomycin, 0.025% soybean trypsin inhibitor (Sigma-Aldrich, St. Louis, MO, USA), and 0.5 mg/ml collagenase (Invitrogen), followed by filtration through a 70-μm cell strainer (BD Bioscience, Franklin Lakes, NJ, USA). The testicular cells were centrifuged in a discontinuous density gradient of Percoll (20, 30, 50, and 60%, GE Healthcare, Little Chalfont, UK) at 25,000 × *g* for 45 min at 4 °C. Leydig cells forming a thick band between the 30 and 50% layers were recovered and seeded onto collagen type I coated dishes. Unattached cells, such as germ line cells, were washed out with phosphate-buffered saline (PBS) 4 h after seeding.

### Knockdown of* Ad4BP/SF-1* and *Srebf2*

For knockdown of *Ad4BP/SF-1*, we used two distinct small interfering RNAs (siRNAs) to exclude a possible off-target effect. Y-1 cells were transfected for 6 h with siRNA duplexes (Mission siRNA: Mm_Nr5a1_1635, 5′-CCUUUAUCUCCAUUGUCGATT-3′ (#1635); Mm_Nr5a1_1636, 5′-CAUUACACGUGCACCGAGATT-3′ (#1636); Sigma-Aldrich) using Lipofectamine RNAiMAX (Invitrogen) for 6 h. The medium was replaced, and the cells were cultured for an additional 48 h. The results obtained with siRNA #1635 are provided in the main text (Fig. 1a, b, Fig. 2c, Fig. 3a, b, and Fig. 4a, d, f), whereas those obtained with siRNA #1636 are provided in Supplementary Fig. 2b, 4b, 6b, 6c, and 8d. A control siRNA (Stealth RNAi Negative Control Medium GC Duplex, Invitrogen) was used as a negative control.

For knockdown of *Ad4BP/SF-1* in Leydig cells, siRNA duplex (stealth RNAi™; Nr5a1-MSS240945, 5′-ACAAGGUGUAAUCCAACAGGGCAGC-3′; Invitrogen) was transfected for 4 h after the cells recovered by Percoll centrifugation were plated. The culture medium was replaced, and the cells were cultured for an additional 39 h. A control siRNA (Stealth RNAi Negative Control Medium GC Duplex, Invitrogen) was used as a negative control.

For the knockdown of *Srebf2*, Y-1 cells were transfected with siRNA duplex (stealth RNAi™; Srebf2-MSS277288, 5′-GGCTTCTTGGCTAGCTACTTCTTAA-3′, Invitrogen) for 6 h, and then cultured for 48 h.

### RNA-seq

Poly-A+ RNA was prepared from si*Ad4BP/SF-1*-treated or siControl-treated Y-1 cells using oligo(dT) magnetic beads. The mRNA-seq library was prepared using NEBNext^®^ Ultra™ Directional RNA Library Prep kit for Illumina^®^ (NEB, Ipswich, MA, USA). After the quality of the library was validated on an Agilent Bioanalyzer 2100 (Agilent Technologies, Santa Clara, CA, USA), the samples were subjected to sequencing with a next-generation sequencer (HiSeq1500, Illumina, San Diego, CA, USA).

Total RNA was prepared from si*Ad4BP/SF-1*-treated or siControl-treated Leydig cells using the RNeasy Mini kit (Qiagen, Hilden, Germany). Then, ribosomal RNAs were removed from the total RNA using the Ribo-zero rRNA removal kit (Illumina). RNA-seq libraries were prepared from the rRNA-depleted RNAs using the Tru-Seq RNA sample prep kit v2 (Illumina). After the library quality was validated on an Agilent Bioanalyzer 2100, the RNA-seq libraries were subjected to sequencing on an Illumina GAIIx. The RNA-seq reads were aligned to the reference mouse genome sequence, UCSC mm10, using TopHat version 2.0.13 with the default parameters^38^. Cufflinks (version 2.2.1) was then used with default parameters to assemble the transcripts and calculate fragments per kilobase of transcript per million mapped fragments^39^.

### Quantitative RT-PCR

Total RNAs were prepared from Y-1 and Leydig cells treated with si*Ad4BP/SF-1* or siControl. cDNAs were synthesized from the RNAs using M-MLV reverse transcriptase (Invitrogen), and then subjected to qRT-PCR. Real-time PCR was performed on a CFX96 instrument (Bio-Rad, Hercules, CA, USA) using SYBR Select master mix (Applied Biosystems). Primers used for qRT-PCR are listed in the Supplementary Data 1. β-Actin (*Actb*) was used as a control.

### Chromatin immunoprecipitation-quantitative PCR

ChIP was performed according to the previously described procedure^9^. Briefly, 2.0 × 10^7^ Y-1 or Leydig cells were fixed with 1% formaldehyde for 5 min at room temperature, and then glycine was added to a final concentration of 125 mM to stop the crosslinking reaction. Then, the fixed cells were lysed with 2 ml ChIP lysis buffer (50 mM Tris-HCl, pH 8.0, 10 mM EDTA, 1% sodium dodecyl sulfate (SDS)), followed by sonication (UCD-300 Bioruptor, Diagenode, Belgium) at 4 °C. After dilution of SDS by addition of 9 volumes of dilution buffer (20 mM Tris-HCl, pH 8.0, 2 mM EDTA, 150 mM NaCl, 1% Triton X-100), the sheared chromatin was subjected to immunoprecipitation with 2 μg of anti-Ad4BP/SF-1 antibody previously produced by ourselves^40^. Normal rabbit IgG was used as a negative control for the immunoprecipitation. The immunoprecipitates were collected using Dynabeads^®^ with Protein A (Life Technologies, Carlsbad, CA, USA), and then sequentially washed three times with 700 μl ChIP-radioimmunoprecipitation assay (RIPA) buffer (50 mM Tris-HCl, pH 8.0, 5 mM EDTA, 150 mM NaCl, 0.1% SDS, 0.5% NP-40), three times with 700 μl high-salt ChIP-RIPA buffer (50 mM Tris-HCl, pH 8.0, 5 mM EDTA, 500 mM NaCl, 0.1% SDS, 0.5% NP-40), three times with 700 μl LiCl buffer (10 mM Tris-HCl, pH 8.0, 1 mM EDTA, 250 mM LiCl, 0.1% NP-40), and once with 700 μl TE buffer (10 mM Tris-HCl, pH 8.0, 1 mM EDTA), all at room temperature. Finally, the chromatin fragments were eluted from the beads with 100 μl elution buffer (0.1 M NaHCO_3_, 1% SDS, 10 mM dithiothreitol). After the crosslinks were reversed by heating at 65 °C for 16 h, DNA fragments were purified using the QIAquick PCR Purification kit (Qiagen). The purified DNAs were subjected to real-time PCR using primer sets listed in Supplementary Data 1. Because ChIP-seq showed that Ad4BP/SF-1 does not bind to exon 7 of the *Star* gene, this region was used as a negative control in ChIP-qPCR.

### ChIP-sequence

The DNA fragments prepared as described above were used to construct a ChIP-seq library using the NEBNext DNA sample prep master mix set 1 (NEB) and DNA Sample Prep kit, oligo only (Illumina). Adaptor-ligated DNA fragments (200–500 bp) were recovered and used for the following steps. After the quality of the library was validated on an Agilent Bioanalyzer 2100 (Agilent Technology), the ChIP-seq library was subjected to sequencing on the Illumina GAIIx platform. ChIP-seq reads were mapped to the reference mouse genome (mm10) using Bowtie^41^ (version 0.12.7), allowing up to three mismatches. Multiple-hit reads were excluded, and only reads uniquely mapped to the reference mouse genome were subjected to further analysis. To identify peaks and regions of Ad4BP/SF-1 enrichment, uniquely mapped reads were analyzed using MACS (Model-based Analysis for ChIP-Seq)^42^ program version 1.4.2 with the option “--nomodel”. The threshold *P* values for enriched regions of Ad4BP/SF-1 were set to 10^−3^.

### Luciferase reporter gene assay

Luciferase reporter genes were constructed using the pGL3 basic vector (Promega, Madison, WI, USA) with upstream regions and the regions where Ad4BP/SF-1 was found to be accumulated by ChIP-seq (ChIP-peak regions). For construction of *Fdps*-Luc, *Fdft1*-Luc, *Msmo1*-Luc, *Dhcr24*-Luc, *Hummer*-Luc, and *Acly*-Luc, the ChIP-peak regions from −6051 to −6631, +27,724 to +28,080, +15,849 to +16,204, −607 to +29, +4581 to +5035, and +13,930 to +14,290 bp, respectively, and 5′ upstream regions from −240 to +50, −311 to +20, −217 to +26, −607 to +29, −1864 to +33, and −1295 to +118 bp, respectively, were used. Structures of the reporters are illustrated in Fig. 2c, Fig. 4d, and Supplemenrary Fig. 8d. Gray, white, and black rectangles represent ChIP-peak regions, promoter regions, and luciferase genes, respectively. Ovals represent locations of potential Ad4BP/SF-1-binding sites^7^. In the *Dhcr24* gene, the ChIP peak overlapped with the promoter region.

Y-1 cells were plated on 24-well plates (Iwaki, Tokyo, Japan) at a density of 1.5 × 10^5^ cells per well. *Fdps*-Luc, *Fdft1*-Luc, *Msmo1*-Luc, and *Dhcr24*-Luc (10 ng) were transfected with si*Ad4BP/SF-1* (#1635) or siControl using Lipofectamine^®^ 2000 transfection reagent (Invitrogen) (Fig. 2c). *Hummr*-Luc and *Acly*-Luc (10 ng) were transfected with si*Ad4BP/SF-1* (#1635), si*Ad4BP/SF-1* (#1636), or siControl into Y-1 cells (Fig. 4d and Supplementary Fig. 6c and 8d). In the experiments shown in Fig. 2d and Supplementary Fig. 8e, 1.5 × 10^5^ HeLa cells were cultured on 24-well plates (Iwaki), and then pGL3 basic vector, *Fdps*-Luc, *Fdft1*-Luc, *Msmo1*-Luc, *Dhcr24*-Luc, and *Acly*-Luc (10 ng) were transfected along with increasing amounts of Ad4BP/SF-1 expression plasmid (25 or 100 ng)^43^. pGL3 basic vector and *Hummr*-Luc (10 ng) were transfected along with increasing amounts of Ad4BP/SF-1 expression plasmid (25, 100, or 300 ng) (Fig. 4e). In Fig. 2e, *Fdps*-Luc, *Msmo1*-Luc, and *Dhcr24*-Luc (10 ng) were transfected into Y-1 cells along with expression plasmids of Ad4BP/SF-1 (300 ng) and transcriptionally active SREBP-2 (1-481) (100 ng)^44^. pCMV-SPORT-βgal (Invitrogen) was used as an internal control. Six hours after transfection, the medium was replaced. Forty-eight hours after transfection, the cells were harvested, and luciferase and β-galactosidase activities were determined.

### Preparation of nuclear extract from Y-1 cells

Y-1 cells (4.0 × 10^7^) were harvested and suspended in 500 μl of hypotonic buffer (10 mM HEPES-KOH, pH 7.9, 1.5 mM MgCl_2_, 10 mM KCl) supplemented with phenylmethylsulfonyl fluoride (PMSF) (Nacalai Tesque, Kyoto, Japan) and benzamidine (Nacalai Tesque). The cells were swelled on ice for 5 min, and then homogenized using a 24-gauge needle (Terumo, Tokyo, Japan). Nuclei were collected by centrifugation at 900 × *g*, for 15 min at 4 °C. The pelleted nuclei were suspended with 200 μl 50 mM HEPES, pH 7.8, 10 mM NaCl, 0.1 mM EDTA, 5 mM MgCl_2_, 2% glycerol, and then the same volume of 50 mM HEPES, pH 7.8, 800 mM NaCl, 0.1 mM EDTA, 5 mM MgCl_2_, 2% glycerol was added. To extract nuclear proteins, the sample was kept on ice for 30 min. Thereafter, the sample was centrifuged (20,000 × *g*, 4 °C, 30 min), and the supernatant was collected as nuclear extract. The nuclear extract was dialyzed overnight with 1 L of binding buffer (see next paragraph for details) supplemented with PMSF and benzamidine, and then used for the subsequent experiments.

### Physical interaction between Ad4BP/SF-1 and SREBP-2

cDNAs encoding full-length Ad4BP/SF-1 or N-terminal region of SREBP-2 (1-481; transcriptionally active form) were cloned into pFastBac plasmids (Thermo Fisher Scientific, Waltham, MA, USA). FLAG-Ad4BP/SF-1, FLAG-SREBP-2, and HA-SREBP-2 were expressed using the Baculovirus Expression System (Thermo Fisher Scientific) in Sf21 cells according to the manufacturer’s protocol. The cells were lysed in 20 mM Tris-HCl (pH 8.0), 400 mM KCl, 10% glycerol, 5 mM MgCl_2_, and 0.1% Tween-20. The FLAG-tagged or HA-tagged proteins were immunopurified with anti-FLAG M2 antibody affinity agarose (Sigma-Aldrich) or anti-HA (clone 12CA5, Abcam, Cambridge, UK) antibody-conjugated agarose beads, and then eluted with 0.25 mg/ml 3× FLAG peptide (Sigma-Aldrich) or with 0.25 mg/ml 3× HA-tag peptide (Cosmo Bio Co., Ltd., Tokyo, Japan), respectively^45,46^. In pull-down experiments (Fig. 2f), FLAG-SREBP-2 (300 ng) was incubated overnight at 4 °C with the nuclear extract prepared from Y-1 cells in 1 ml binding buffer (50 mM HEPES-KOH, pH 7.4, 150 mM NaCl, 1 mM EDTA, 0.1% NP-40) supplemented with PMSF and benzamidine. FLAG-SREBP-2 (1-481) and its interactants were immunoprecipitated with anti-FLAG antibody-conjugated magnetic beads (Sigma-Aldrich), and then the beads were sequentially washed three times with 700 μl low-salt washing buffer (50 mM HEPES-KOH, pH 7.4, 150 mM NaCl, 0.1% NP-40), three times with 700 μl high-salt washing buffer (50 mM HEPES-KOH, pH 7.4, 300 mM NaCl, 0.1% NP-40), and once with 700 μl PBS. Finally, the immunoprecipitates were eluted from the beads with 15 μl elution buffer (50 mM Tris-HCl, pH 7.4, 150 mM NaCl, 500 μg/ml FLAG peptide). Eluates and inputs were subjected to SDS-polyacrylamide gel electrophoresis followed by western blotting using antibodies against Ad4BP/SF-1 (1/1000), FLAG (1/1000, Nacalai Tesque), or CREB (cAMP-responsive element binding protein, 1/1000, Millipore, Billerica, MA, USA). CREB was used as a negative control. For in vitro binding assays (Fig. 2g), HA-SREBP-2 (1-481) (300 ng) and FLAG-Ad4BP/SF-1 (300 ng) were incubated overnight at 4 °C in 1 ml binding buffer. Following the same washing and elution procedures described for the pull-down experiment above, western blotting was performed using antibody against HA (1/1000) or FLAG (1/1000).

### Measurement of de novo cholesterol synthetic activity

De novo cholesterogenic activity was measured as described previously^47,48^. Y-1 cells were treated with si*Ad4BP/SF-1* (#1635) or siControl for 48 h, and then incubated for 1 h at 37 °C in a serum-free medium containing [1,2-^14^C]acetate (PerkinElmer, Boston, MA, USA), 50 μM aminoglutethimide (Sigma-Aldrich) and 2 μg/ml 58-035 (ACAT2; acyl-CoA cholesterol acyltransferase inhibitor) (Sigma-Aldrich). Total lipid fraction containing cholesterol was extracted with chloroform/methanol (2:1, v/v), and then separated by thin-layer chromatography on a silica gel using benzene-ethylacetate (2:3, v/v) as a solvent. The radioactivities of the spots for free and esterified cholesterol, visualized with iodine vapor, were determined by liquid scintillation counting. [1,2-^14^C]acetate incorporation into cholesterol was expressed as cpm/mg protein/h.

### Quantification of cholesterol and related metabolites

Y-1 cells were treated with si*Ad4BP/SF-1* (#1635) or siControl for 48 h, and then whole cells or mitochondria isolated from the cells were suspended in 0.2 ml methanol at 4 °C. The suspension was sonicated (2 × 20 s pulses, separated by a 30-s interval) on a Bioruptor^®^ Plus (Diagenode). Thereafter, they were centrifuged at 15,000 rpm for 5 min at 4 °C to eliminate methanol-insoluble cellular components. The supernatant (methanol soluble fraction) was recovered and evaporated to remove methanol. Gas chromatography-mass spectrometry analysis was performed using an Agilent 6890 Plus gas chromatograph interfaced with a single-quadrupole Agilent 5975C MSD (Agilent) as previously described^49^.

### Statistics

All experiments were performed in at least three biologically independent replicates. Data are represented as average and standard deviation (SD). The numbers of the experimental replicates are indicated with “*n*” in figure legends. Statistical significance was examined using a two-tailed Student’s *t* test.

### Data availability

mRNA-seq data have been deposited in DDBJ under the accession code DRA005961 (Y-1 cells) and DRA005963 (Leydig cells) (http://trace.ddbj.nig.ac.jp/DRASearch/).

ChIP-seq data have been deposited in GEO under the accession code GSE106955 (https://www.ncbi.nlm.nih.gov/geo/).

## Electronic supplementary material

Supplementary Information

Description of Additional Supplementary Files

Supplementary Data 1
