# Supplementary material for: Ad4BP/SF-1 regulates cholesterol synthesis to boost the production of steroids
Source: Commun Biol. 2018 Mar 22;1:18. doi: 10.1038/s42003-018-0020-z (PMC6123728; doi:10.1038/s42003-018-0020-z)
Supplement: Supplementary file 1 — Supplementary Information [file 42003_2018_20_MOESM1_ESM.pdf]

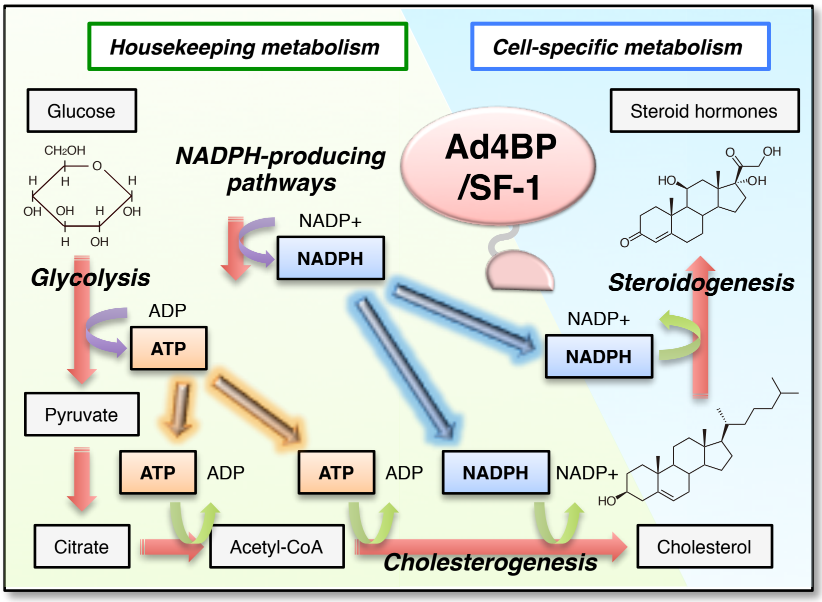

Supplementary Figure 1. Orchestration of housekeeping and cell-specific metabolism by Ad4BP/SF-1

Ad4BP/SF-1 regulates housekeeping glycolysis and cholesterogenesis in addition to cell-specific steroidogenesis, and thereby governs these metabolic pathways as a combined regulatory unit. ATP, necessary for acetyl-CoA production and cholesterogenesis, is produced by glycolysis. Not all but some of the major pathways producing NADPH, necessary for cholesterogenesis and steroidogenesis, are regulated by Ad4BP/SF-1. Thus, production and consumption of ATP and NADPH are coordinated through the involvement of Ad4BP/SF-1 in these metabolic pathways. Red arrows indicate reactions regulated by Ad4BP/SF-1. Orange and blue arrows indicate supply routes for ATP and NADPH, respectively.

a

Gene name	Y-1 cells FPKM Control	Y-1 cells FPKM *Ad4BP* KD	Ratio
Acat2	68.0	35.0	0.51
Hmgcs1	267.3	138.7	0.52
Hmgcr	131.1	66.0	0.50
Mvk	38.2	29.1	0.76
Pmvk	46.3	30.2	0.65
Mvd	94.2	40.1	0.43
Fdps	467.2	233.7	0.50
Idi1	171.6	100.8	0.59
Fdft1	67.2	39.3	0.58
Sqle	221.2	111.6	0.50
Lss	56.7	27.9	0.49
Cyp51	131.9	67.7	0.51
Tm7sf2	38.0	43.3	1.14
Msmo1	381.8	218.0	0.57
Nsdhl	72.6	49.9	0.69
Hsd17b7	25.7	23.4	0.91
Ebp	48.9	37.6	0.77
Sc5d	110.6	60.6	0.55
Dhcr7	101.7	46.9	0.46
Dhcr24	136.1	93.3	0.69

1/3 — 3.0

b

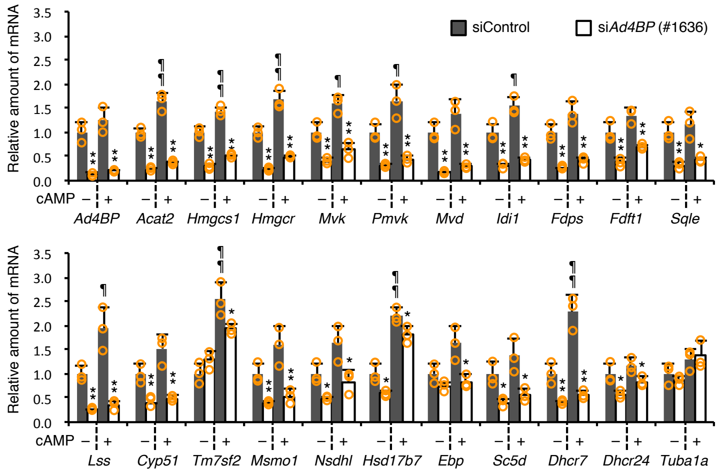

Supplementary Figure 2. Cholesterogenic gene expression in Y-1 cells affected by Ad4BP/SF-1 knockdown

a, Cholesterogenic gene expression in Y-1 cells determined by mRNA-seq is shown. FPKM values and ratios of the gene expression (*Ad4BP/SF-1* knockdown vs. Control) are indicated. Magenta and turquoise indicate up- and down-regulated expression, respectively. ***b,*** Cholesterogenic gene expression in *Ad4BP/SF-1* knockdown and control Y-1 cells was determined by qRT-PCR in the presence (+) or absence (-) of cAMP. To exclude an off-target effect, another si*Ad4BP/SF-1* (#1636) was used. Average values and SDs are indicated. The average values for the siControl-treated cells in the absence of cAMP were normalized to 1.0. **p* <0.05, ***p* <0.01. Bars with "¶" are compared to vehicle-treated cells; ¶*p* <0.05, ¶¶*p* <0.01, n=3.

a

Leydig cells			
Gene name	FPKM		Ratio
	Control	*Ad4BP* KD	
Acat2	48.4	49.9	1.03
Hmgcs1	211.9	233.5	1.10
Hmgcr	52.0	77.6	1.49
Mvk	21.3	25.0	1.18
Pmvk	20.3	19.0	0.93
Mvd	16.8	19.7	1.17
Fdps	379.1	252.3	0.67
Idi1	66.3	95.4	1.44
Fdft1	62.5	55.7	0.89
Sqle	69.9	74.0	1.06
Lss	30.5	36.7	1.21
Cyp51	90.2	94.3	1.04
Tm7sf2	26.9	28.2	1.05
Msmo1	13.1	7.7	0.59
Nsdhl	29.9	33.1	1.10
Hsd17b7	29.8	33.3	1.12
Ebp	27.0	29.1	1.08
Sc5d	144.0	101.8	0.71
Dhcr7	29.4	17.6	0.60
Dhcr24	185.5	151.5	0.82

cAMP-treated Leydig cells			
Gene name	FPKM		Ratio
	Control	*Ad4BP* KD	
Acat2	139.0	114.0	0.82
Hmgcs1	562.4	354.9	0.63
Hmgcr	167.9	116.4	0.69
Mvk	48.5	34.8	0.72
Pmvk	79.5	34.6	0.44
Mvd	52.8	25.4	0.48
Fdps	816.6	370.5	0.45
Idi1	283.5	227.0	0.80
Fdft1	148.1	99.6	0.67
Sqle	169.2	126.8	0.75
Lss	105.5	59.3	0.56
Cyp51	285.9	160.7	0.56
Tm7sf2	41.2	30.4	0.74
Msmo1	11.1	6.1	0.54
Nsdhl	63.5	44.9	0.71
Hsd17b7	105.8	47.3	0.45
Ebp	42.5	41.2	0.97
Sc5d	611.1	243.5	0.40
Dhcr7	74.3	20.7	0.28
Dhcr24	317.9	210.6	0.66

1/3 3.0

b

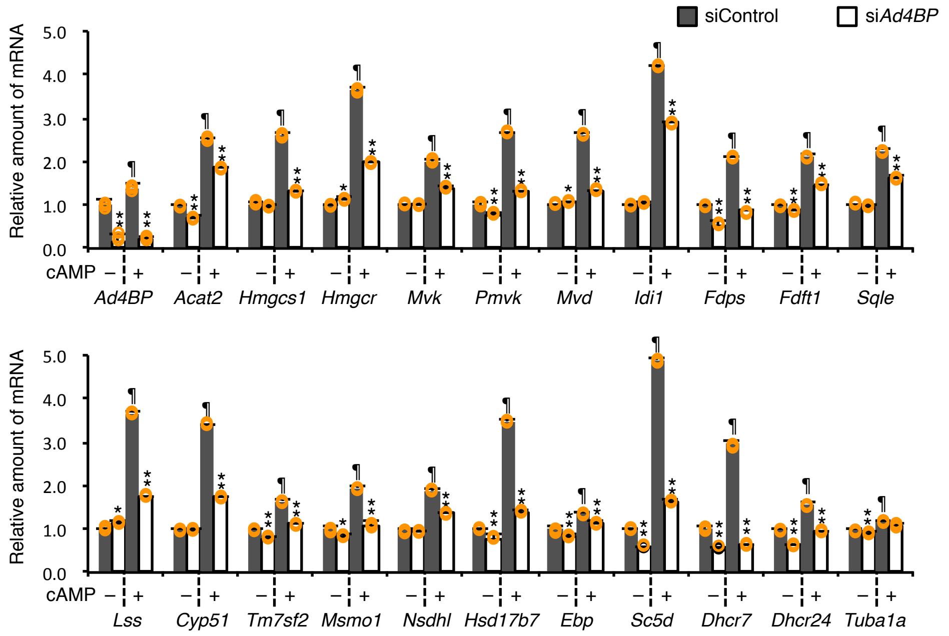

Supplementary Figure 3. Cholesterogenic gene expression in Leydig cells affected by Ad4BP/SF-1 knockdown

a, Cholesterogenic gene expression in Leydig cells determined by mRNA-seq in the presence (right) or absence (left) of cAMP is shown. FPKM values and ratios of the gene expressions (*Ad4BP/SF-1* knockdown vs. Control) are indicated. Colorimetric

indication is same as in Supplementary Figure 2. ***b,*** Cholesterogenic gene expression in *Ad4BP/SF-1* knockdown and control Leydig cells in the presence (+) or absence (-) of cAMP was determined by qRT-PCR. Average values and SDs are indicated. The average values for the siControl-treated cells in the absence of cAMP were normalized to 1.0. Bars with "*" are compared to siControl-treated cells; *p <0.05, **p <0.01. Bars with an "¶" are compared to vehicle-treated cells; ¶p <0.05, ¶¶p <0.01, n=3.

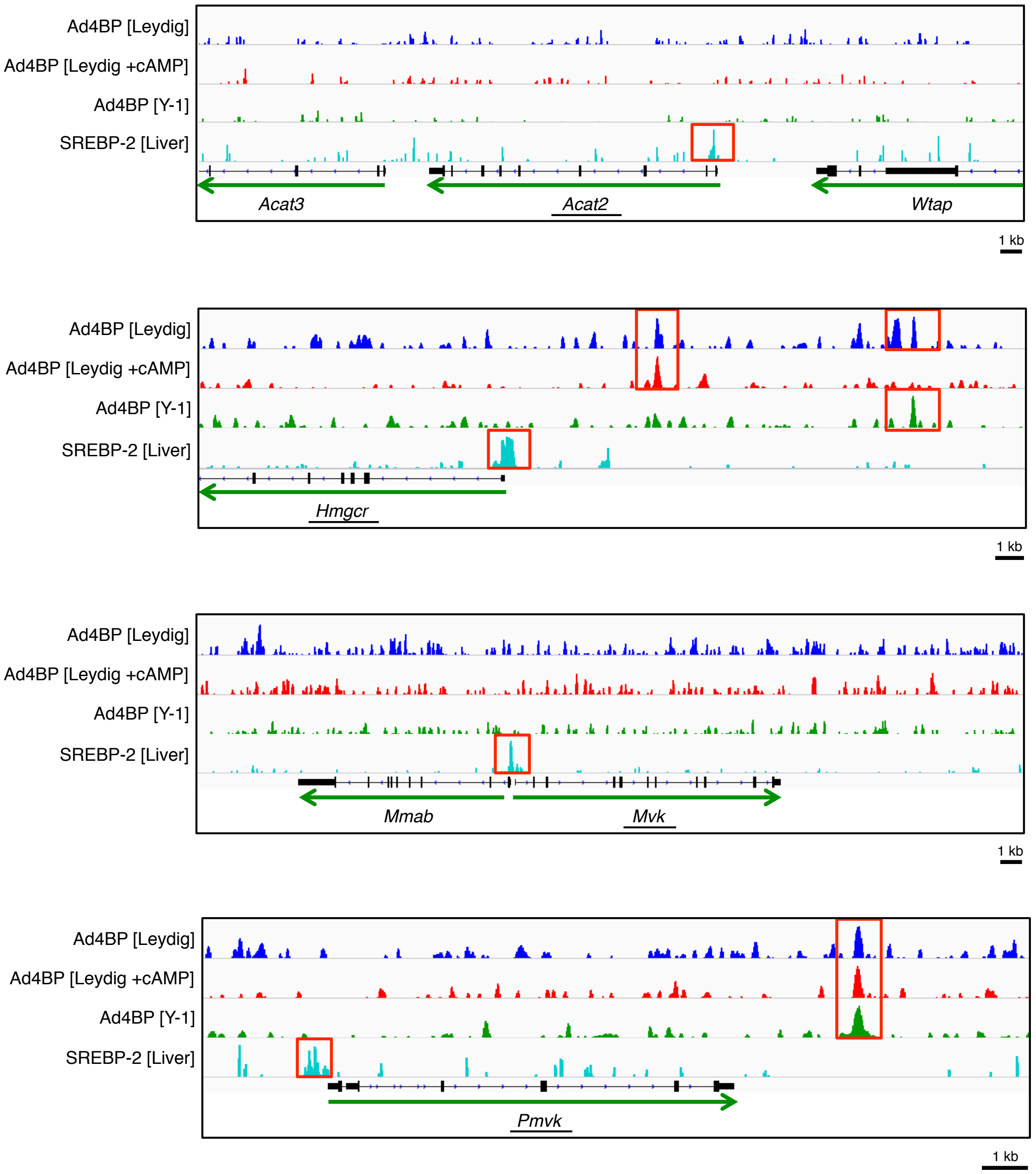
Ad4BP [Leydig]
Ad4BP [Leydig +cAMP]
Ad4BP [Y-1]
SREBP-2 [Liver]
Acat3
Acat2
Wtap
1 kb
Ad4BP [Leydig]
Ad4BP [Leydig +cAMP]
Ad4BP [Y-1]
SREBP-2 [Liver]
Hmgcr
1 kb
Ad4BP [Leydig]
Ad4BP [Leydig +cAMP]
Ad4BP [Y-1]
SREBP-2 [Liver]
Mmab
Mvk
1 kb
Ad4BP [Leydig]
Ad4BP [Leydig +cAMP]
Ad4BP [Y-1]
SREBP-2 [Liver]
Pmvk
1 kb

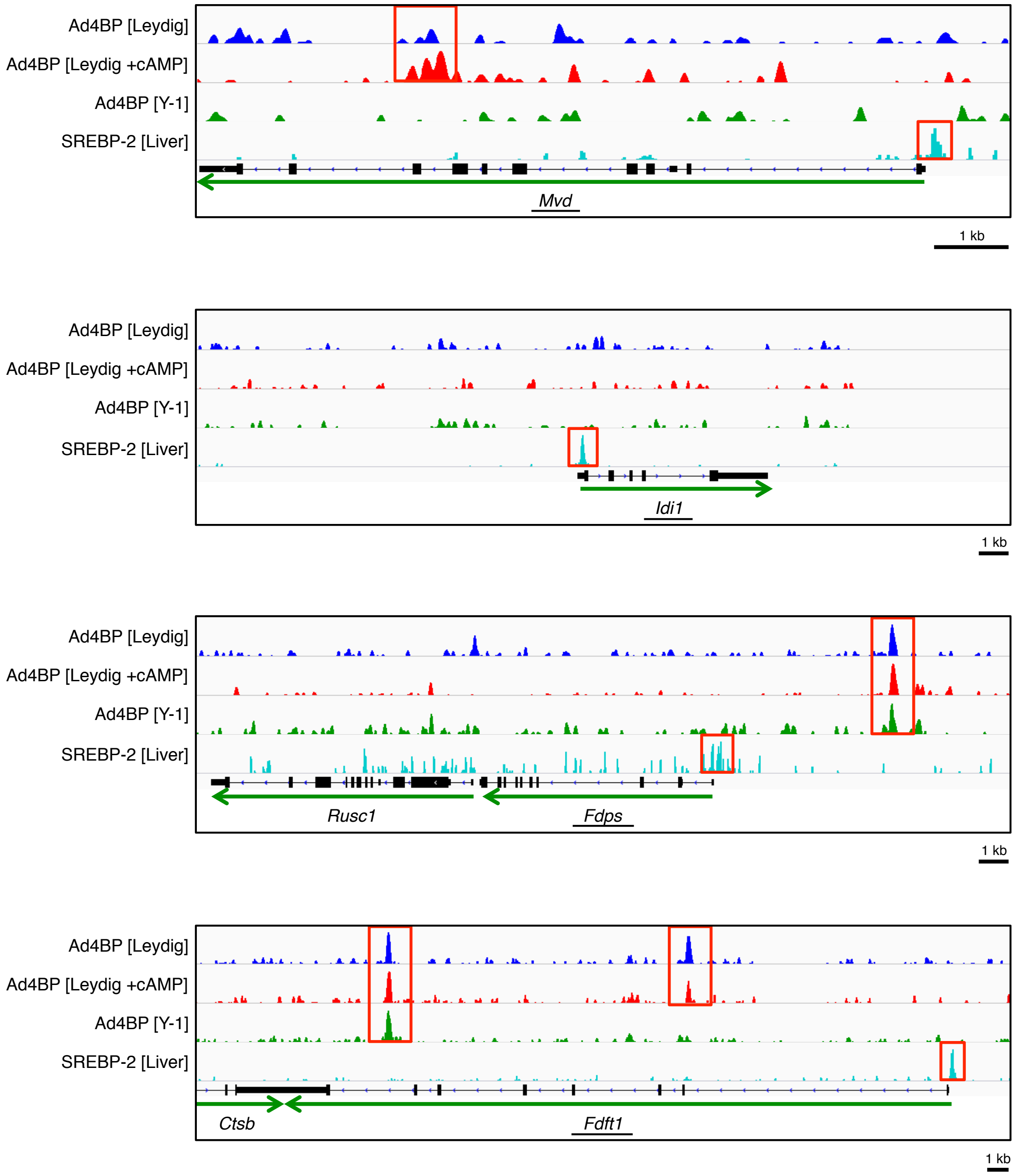

Ad4BP [Leydig]
Ad4BP [Leydig +cAMP]
Ad4BP [Y-1]
SREBP-2 [Liver]
Mvd
1 kb
Ad4BP [Leydig]
Ad4BP [Leydig +cAMP]
Ad4BP [Y-1]
SREBP-2 [Liver]
Idi1
1 kb
Ad4BP [Leydig]
Ad4BP [Leydig +cAMP]
Ad4BP [Y-1]
SREBP-2 [Liver]
Rusc1
Fdps
1 kb
Ad4BP [Leydig]
Ad4BP [Leydig +cAMP]
Ad4BP [Y-1]
SREBP-2 [Liver]
Ctsb
Fdft1
1 kb

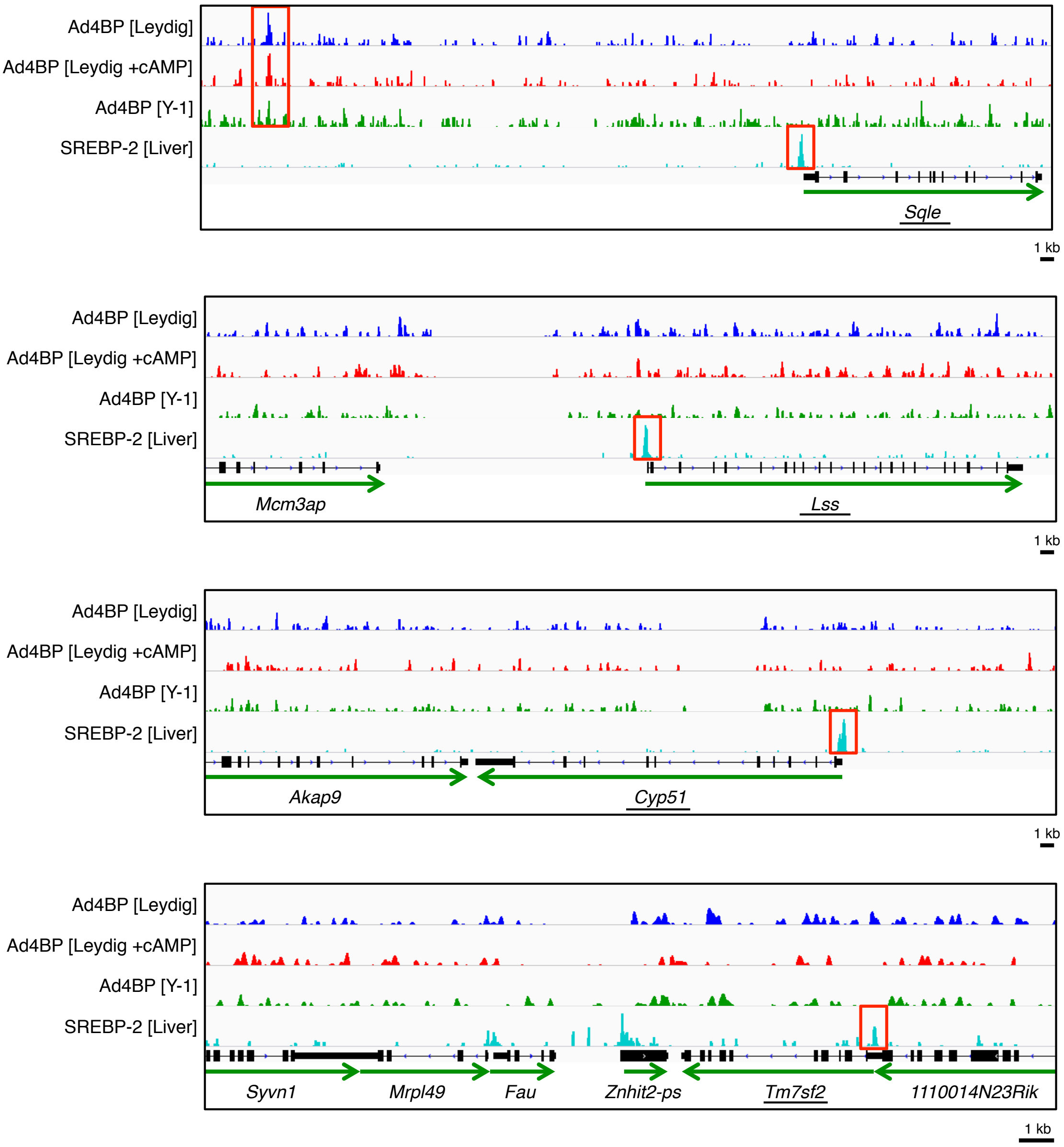
Ad4BP [Leydig]
Ad4BP [Leydig +cAMP]
Ad4BP [Y-1]
SREBP-2 [Liver]
Sqle
1 kb
Mcm3ap
Lss
Akap9
Cyp51
Syvn1
Mrpl49
Fau
Znhit2-ps
Tm7sf2
1110014N23Rik

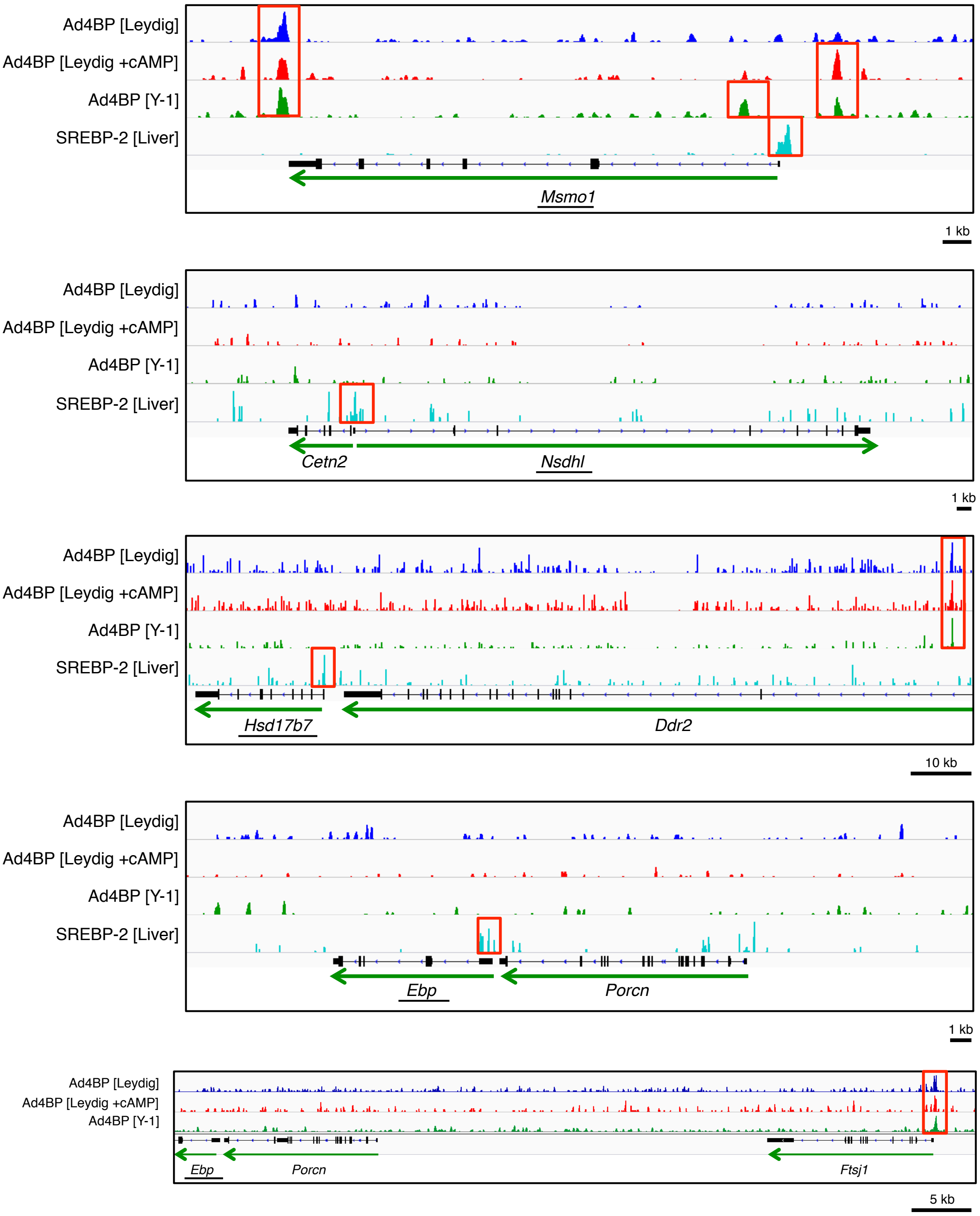
Ad4BP [Leydig]
Ad4BP [Leydig +cAMP]
Ad4BP [Y-1]
SREBP-2 [Liver]
Msmo1
1 kb
Ad4BP [Leydig]
Ad4BP [Leydig +cAMP]
Ad4BP [Y-1]
SREBP-2 [Liver]
Cetn2
Nsdhl
1 kb
Ad4BP [Leydig]
Ad4BP [Leydig +cAMP]
Ad4BP [Y-1]
SREBP-2 [Liver]
Hsd17b7
Ddr2
10 kb
Ad4BP [Leydig]
Ad4BP [Leydig +cAMP]
Ad4BP [Y-1]
SREBP-2 [Liver]
Ebp
Porcn
1 kb
Ad4BP [Leydig]
Ad4BP [Leydig +cAMP]
Ad4BP [Y-1]
Ebp
Porcn
Ftsj1
5 kb

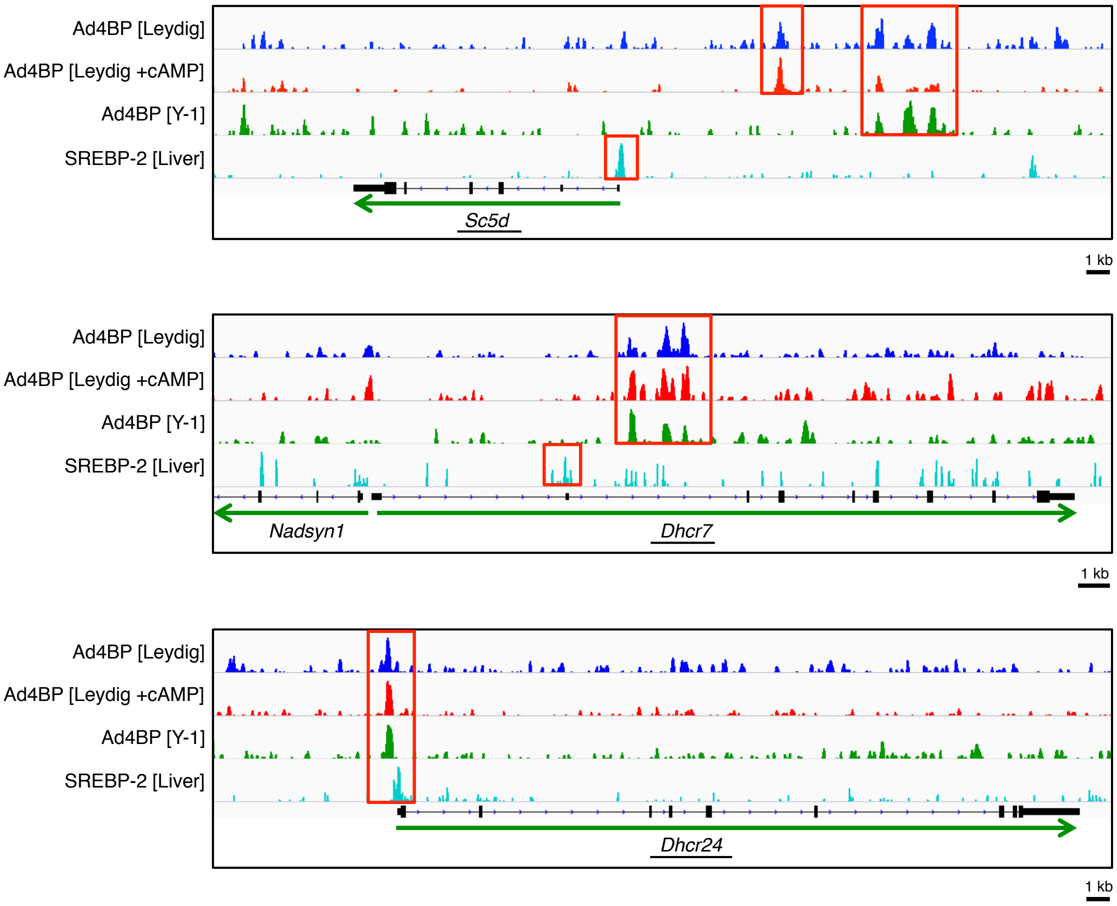

Supplementary Figure 4. Binding of Ad4BP/SF-1 and SREBP-2 to cholesterogenic gene loci

Accumulation of Ad4BP/SF-1 and SREBP-2 on the cholesterogenic genes revealed by ChIP-seq studies is shown. Blue, red, and green histograms depict accumulation of Ad4BP/SF-1 in Leydig, cAMP-treated Leydig, and Y-1 cells, respectively. Light blue histogram indicating accumulation of SREBP-2 in the mouse liver was depicted based on the data reported previously[13]. Black boxes indicate exons. Green arrows indicate directions of gene transcription. The ChIP peaks discussed in the text are enclosed by red squares.

a

Cell	FPKM values of *Srebf2*		Ratio
	Control	*Ad4BP* KD	
Y-1	147.2	117.8	0.80
Leydig	20.7	22.9	1.11
Leydig +cAMP	31.6	26.1	0.83

2/3 — 1.5

b

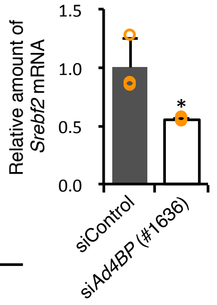

c

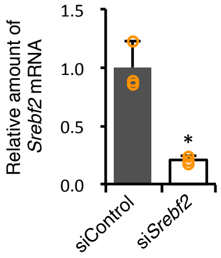

d

Gene name	FPKM		Ratio
	Control	*Srebf2* KD	
Acat2	68.0	29.8	0.44
Hmgcs1	267.3	86.1	0.32
Hmgcr	131.1	55.0	0.42
Mvk	38.2	14.1	0.37
Pmvk	46.3	28.9	0.62
Fdps	467.2	247.1	0.53
Idi1	171.6	54.9	0.32
Fdft1	67.2	32.6	0.49
Sqle	221.2	69.9	0.32
Lss	56.7	24.9	0.44
Cyp51	131.9	48.2	0.37
Tm7sf2	38.0	17.8	0.47
Msmo1	381.8	139.3	0.36
Nsdhl	72.6	16.3	0.22
Hsd17b7	25.7	4.1	0.16
Ebp	48.9	24.6	0.50
Sc5d	110.6	61.2	0.55
Dhcr7	101.7	55.7	0.55
Dhcr24	136.1	76.7	0.56

1/3 — 3.0

Supplementary Figure 5. Cholesterogenic gene expression decreased by Srebf2 knockdown

a, *Srebf2* expression (FPKM values) and ratios of the gene expression (*Ad4BP/SF-1* knockdown vs. Control) in Y-1, Leydig, and cAMP-treated Leydig cells revealed by mRNA-seq are shown. Colorimetric indication is the same as in Supplementary Figure 2. ***b,*** Expression of *Srebf2* in si*Ad4BP/SF-1* (#1636)–treated Y-1 cells was determined by qRT-PCR. Average values and SDs of *Srebf2* expression are indicated. The average value for the siControl-treated cells was normalized to 1.0. **p* <0.05. n=3. ***c,*** Knockdown of *Srebf2* by siRNA treatment was evaluated by qRT-PCR in Y-1 cells. Average values and SDs of *Srebf2* expression are indicated. The average value for the siControl-treated cells was normalized to 1.0. **p* <0.05. n=3. ***d,*** FPKM values and ratios

of cholesterogenic gene expression (*Srebf2* knockdown vs. Control) in Y-1 cells are shown. Colorimetric indication in ***a*** and ***d*** is same as in Supplementary Figure 2.

a

Y-1 cells			
Gene name	FPKM		Ratio
	Control	*Ad4BP* KD	
Ldlr	62.2	35.0	0.56
Scarb1	109.0	66.8	0.61
Abca1	6.0	6.4	1.07
Abcg1	0.0	0.0	1.00

Leydig cells			
Gene name	FPKM		Ratio
	Control	*Ad4BP* KD	
Ldlr	35.9	39.8	1.11
Scarb1	63.6	36.9	0.58
Abca1	174.3	154.3	0.89
Abcg1	2.4	2.3	0.96

cAMP-treated Leydig cells			
Gene name	FPKM		Ratio
	Control	*Ad4BP* KD	
Ldlr	70.3	51.4	0.73
Scarb1	157.8	34.9	0.22
Abca1	223.9	262.1	1.17
Abcg1	0.8	0.9	1.13

1/3 3.0

b

Cell type	FPKM values of *Hummr*		Ratio
	Control	*Ad4BP* KD	
Y-1	182.4	74.1	0.41
Leydig	900.6	249.6	0.28
Leydig +cAMP	430.9	227.3	0.53

1/3 3.0

c

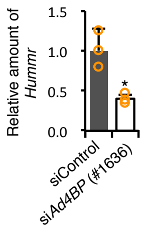

d

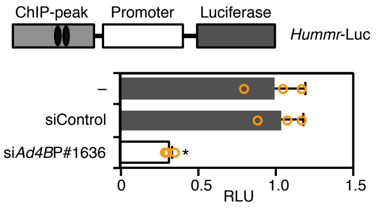

Supplementary Figure 6. Down-regulation of genes related to cholesterol uptake and transport by Ad4BP/SF-1 knockdown

a, Expression of genes involved in cholesterol uptake and excretion (FPKM values) revealed by mRNA-seq and ratios of the gene expression (*Ad4BP/SF-1* knockdown vs. Control) in Y-1, Leydig, and cAMP-treated Leydig cells are shown. ***b,*** *Hummr* gene expression in control and *Ad4BP/SF-1* knockdown Y-1, Leydig, and cAMP-treated Leydig cells was determined by mRNA-seq. The expression (FPKM values) and ratios of the gene expression (*Ad4BP/SF-1* knockdown vs. Control) are shown. Colorimetric indication in ***a*** and ***b*** is the same as in Supplementary Figure 2. ***c,*** Expression of *Hummr* was examined in *Ad4BP/SF-1* knockdown (#1636) Y-1 cells by qRT-PCR. Average values and SDs of *Hummr* expression are indicated. The average value for the siControl-treated cells was normalized to 1.0. *p <0.05. n=3. ***d,*** *Hummr*-Luciferase reporter activity was examined in control and *Ad4BP/SF-1* knockdown (#1636) cells. *p <0.05. n=3.

a

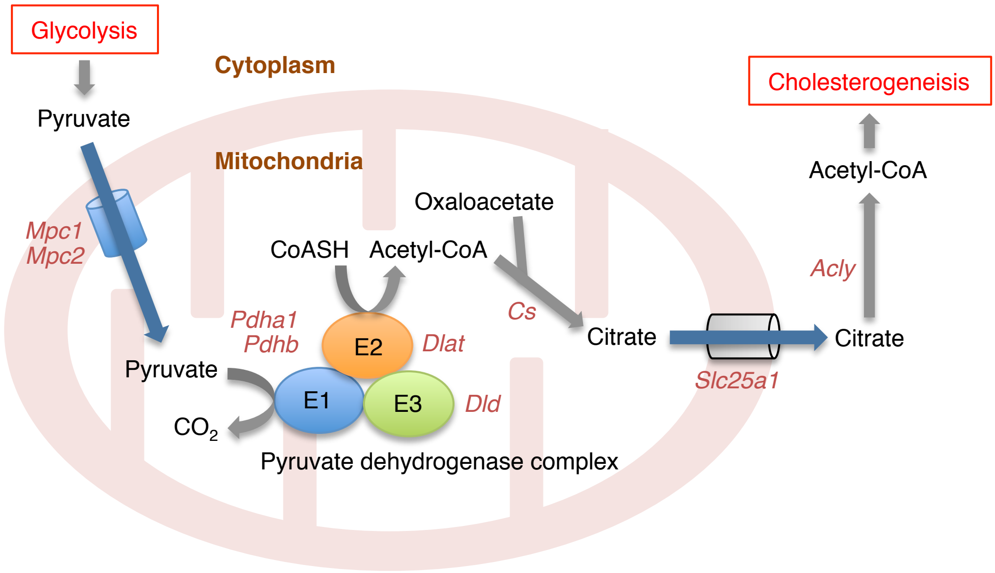

b

Y-1 cells			
Gene name	FPKM		Ratio
	Control	*Ad4BP* KD	
Mpc1	57.0	54.8	0.96
Mpc2	64.4	45.3	0.70
Pdha1	43.3	51.5	1.19
Pdhb	82.2	65.7	0.80
Dlat	28.1	20.1	0.72
Dld	78.5	50.1	0.64
Cs	143.6	115.3	0.80
Slc25a1	68.5	53.9	0.79
Acly	246.7	162.2	0.66

Leydig cells			
Gene name	FPKM		Ratio
	Control	*Ad4BP* KD	
Mpc1	81.9	58.0	0.71
Mpc2	61.7	75.5	1.22
Pdha1	39.6	46.8	1.18
Pdhb	56.2	38.9	0.69
Dlat	13.0	10.1	0.78
Dld	50.6	46.4	0.92
Cs	43.1	41.0	0.95
Slc25a1	37.1	28.7	0.77
Acly	122.8	104.9	0.85

cAMP-treated Leydig cells			
Gene name	FPKM		Ratio
	Control	*Ad4BP* KD	
Mpc1	80.9	58.3	0.72
Mpc2	81.5	94.6	1.16
Pdha1	44.2	50.9	1.15
Pdhb	48.8	38.6	0.79
Dlat	12.5	9.9	0.80
Dld	50.0	51.0	1.02
Cs	43.9	44.2	1.01
Slc25a1	61.8	41.3	0.67
Acly	275.2	131.1	0.48

1/3 3.0

Supplementary Figure 7. Regulation of genes involved in cholesterol uptake and conversion of pyruvate to acetyl-CoA

a, Whole process of conversion of the end product of glycolysis, pyruvate, to the starting material of cholesterogenesis, acetyl-CoA, is shown. Genes involved in this process are indicated. ***b,*** Expression of the genes above (FPKM values) revealed by mRNA-seq and ratios of the gene expression (*Ad4BP/SF-1* knockdown vs. Control) in Y-1, Leydig, and cAMP-treated Leydig cells are shown. Colorimetric indication is the same as in Supplementary Figure 2.

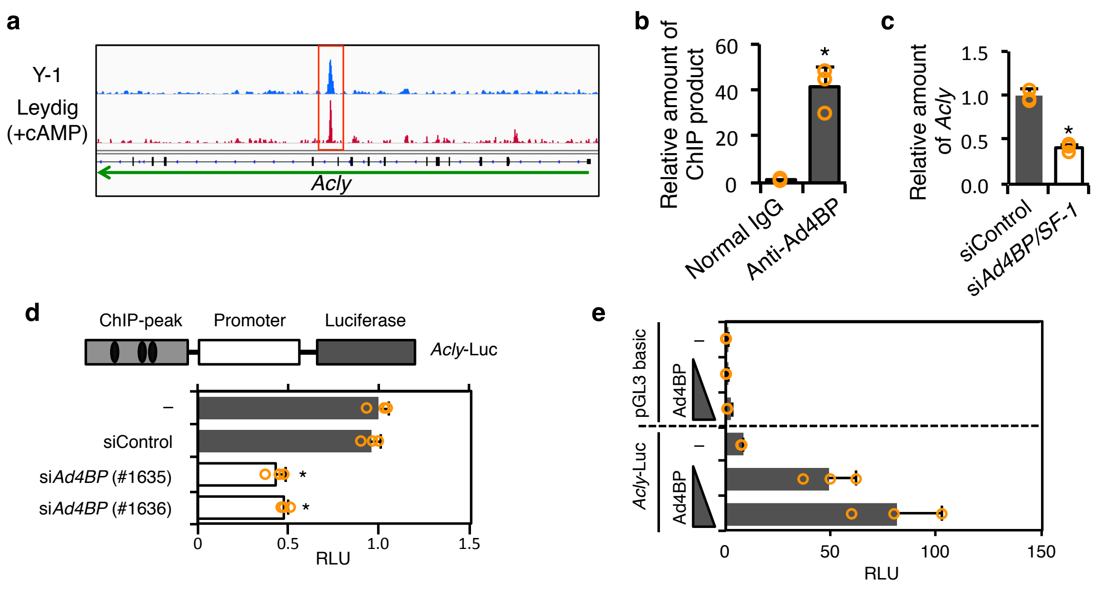

Supplementary Figure 8. Possible direct regulation of the Acly gene by Ad4BP/SF-1

a, Accumulation of Ad4BP/SF-1 in Y-1 (Blue histogram) and cAMP-treated Leydig cells (red histogram) revealed by ChIP-seq are shown for the *Acly* gene. The ChIP peaks are enclosed by red squares. ***b,*** Accumulation of Ad4BP/SF-1 to *Acly* was validated by ChIP-qPCR. Average values and SDs are shown. The average value of the control was normalized to 1.0. *p <0.005. n=3. ***c,*** Expression of *Acly* was examined in *Ad4BP/SF-1* knockdown Y-1 cells by qRT-PCR. Average values and SDs of *Acly* expression are indicated. The average value for the siControl-treated cells was normalized to 1.0. *p <0.005. ***d,*** A luciferase reporter gene construct, *Acly*-Luc, and reporter activity in control and *Ad4BP/SF-1* knockdown Y-1 cells. *p <0.05. ***e,*** Reporter gene activity was examined in the presence of overexpressed *Ad4BP/SF-1* in HeLa cells. ***d and e,*** Average values and SDs of luciferase activities. Average value with siControl treatment (***d***) or that for pGL3basic without *Ad4BP/SF-1* overexpression (***e***) was normalized to 1.0. n=3.

a

Gene name	FPKM		
	Progenitor cells	Fetal Leydig cells	Ratio
Ad4BP/SF-1	49.9	850.2	17.03
Srebf2	86.7	151.9	1.75
Acat2	32.7	148.1	4.53
Hmgcs1	179.9	888.8	4.94
Hmgcr	82.1	344.5	4.19
Mvk	22.0	187.9	8.56
Pmvk	31.4	230.4	7.33
Mvd	19.1	289.8	15.21
Fdps	193.5	2624.1	13.56
Idi1	78.3	281.8	3.60
Fdft1	51.2	216.9	4.23
Sqle	136.8	313.9	2.29
Lss	23.9	316.8	13.28
Cyp51	86.1	354.7	4.12
Tm7sf2	27.7	629.5	22.71
Msmo1	101.2	696.7	6.88
Nsdhl	37.3	281.0	7.53
Hsd17b7	19.5	76.1	3.91
Ebp	11.0	150.7	13.68
Sc5d	23.3	106.5	4.57
Dhcr7	42.3	750.4	17.76
Dhcr24	59.9	126.8	2.11
Hummr/Mgarp	2.9	134.1	46.24
Acly	75.5	456.3	6.04

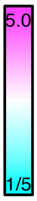

b

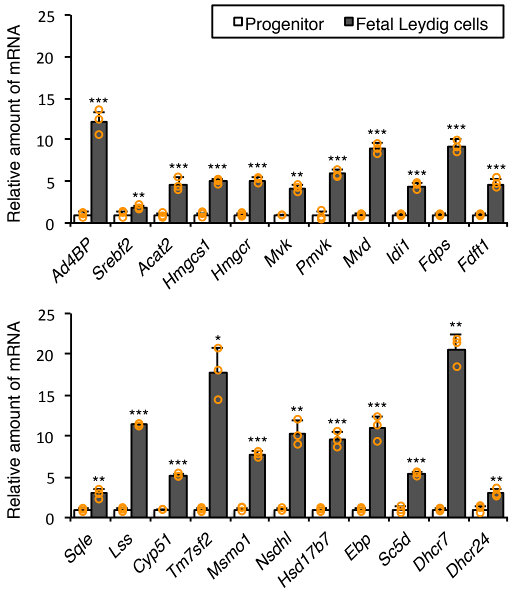

Supplementary Figure 9. UP-regulation of cholesterogenic genes during differentiation of mouse fetal Leydig cells

a, Expression of *Ad4BP/SF-1*, *Srebf2*, cholesterogenic genes, and *Hummr* were examined with mouse fetal Leydig and progenitor cells. FPKM (fragments per kilobase of transcript per million mapped fragment) values and the ratios (fetal Leydig vs. progenitor cells) are indicated. Magenta and turquoise indicate increased and decreased gene expression, respectively. ***b,*** Cholesterogenic gene expression in fetal Leydig and progenitor cells were determined by qRT-PCR. Average values and SDs are indicated. The average values for the progenitor cells were normalized to 1.0. **p* <0.05, ***p* <0.01. ****p* <0.005. n=3.

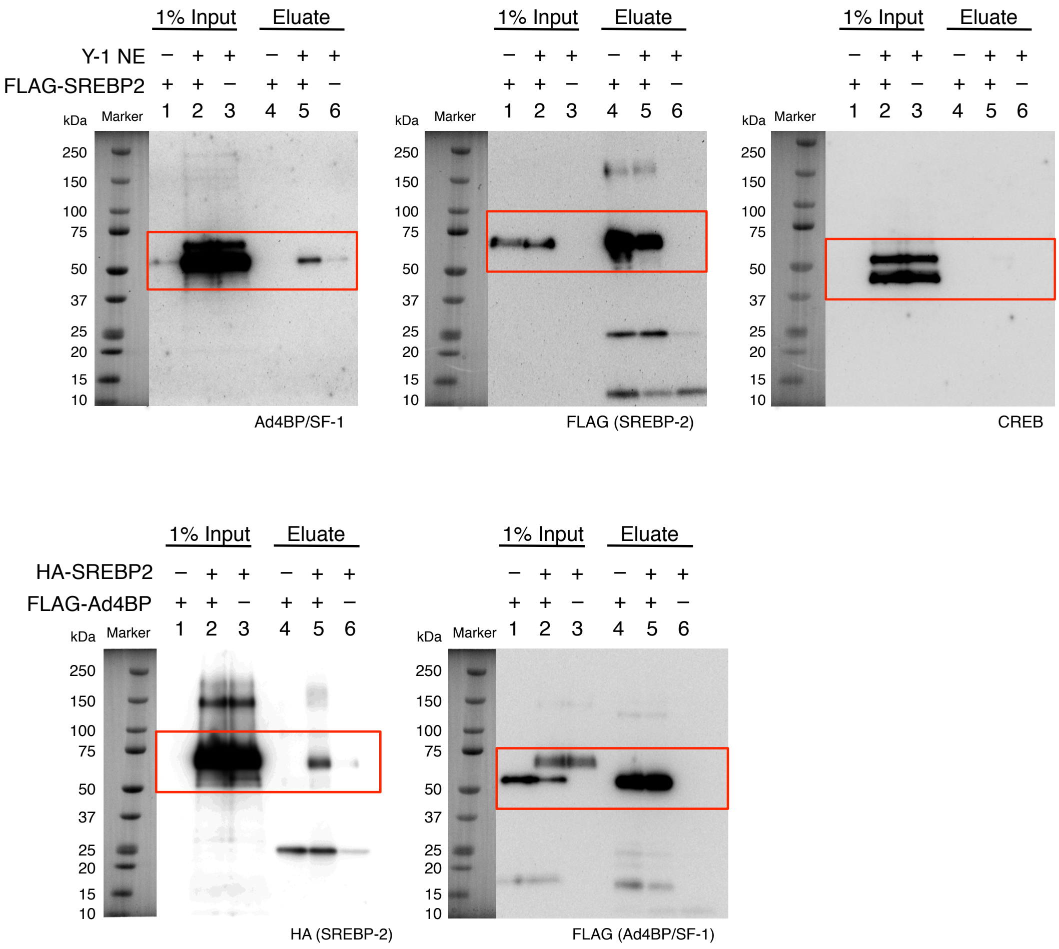

Supplementary Figure 10. Full blot images for cropped gels.

The boxed regions are used in Fig. 3b and c.

Supplementary Table 1.

Accumulation of Ad4BP/SF-1 to cholesterogenic gene loci, as revealed by ChIP-seq with Y-1 and cAMP-treated Leydig cells. The presence or absence of ChIP peaks and their positions are shown. Numbers of consensus Ad4BP/SF-1-binding sites in ChIP-peak regions and conservation of these sites in the human genome are also indicated.

Gene name	Peak position (Distance from TSS)	Y-1	Leydig (cAMP)	Number of Ad4 sites in Mouse	Ad4 site in Human
Acat2	–	–	–	–	–
Hmgcs1	Not applicable	–	–	–	–
Hmgcr	Upstream (5.0 kb)	×	○	1	○
	Upstream (14 kb)	○	×	1	○
Mvk	–	–	–	–	–
Pmvk	Downstream (15 kb)	○	○	1	○
Mvd	Intron 7 (6.7 kb)	×	○	1	○
Idi1	–	–	–	–	–
Fdps	Upstream (6.0 kb)	○	○	1	○
Fdft1	Intron 1 (13 kb)	×	○	2	○
	Intron 7 (28 kb)	○	○	2	○
Sqle	Upstream (35 kb)	○	○	1	○
Lss	–	–	–	–	–
Cyp51	–	–	–	–	–
Tm7sf2	–	–	–	–	–
Msmo1	Upstream (2.0 kb)	○	○	2	○
	Intron 1 (1.0 kb)	○	×	1	○
	Downstream (15 kb)	○	○	1	○
Nsdhl	–	–	–	–	–
Hsd17b7	Upstream (95 kb)	○	○	1	○
Ebp	Upstream (55 kb)	○	○	2	○
Sc5d	Upstream (12 kb)	○	○	1	○
	Upstream (11 kb)	○	○	2	○
	Upstream (10 kb)	○	○	2	○
	Upstream (6.0 kb)	×	○	2	○
Dhcr7	Intron 1 (2.3 kb)	○	○	1	○
	Intron 1 (3.6 kb)	○	○	4	○
	Intron1 (4.2 kb)	○	○	1	○
Dhcr24	Basal promoter	○	○	1	○
