# Supplementary material for: Ad4BP/SF-1 regulates cholesterol synthesis to boost the production of steroids
Source: Commun Biol. 2018 Mar 22;1:18. doi: 10.1038/s42003-018-0020-z (PMC6123728; doi:10.1038/s42003-018-0020-z)
Supplement: Supplementary file 2 — Description of Additional Supplementary Files [file 42003_2018_20_MOESM2_ESM.docx]

**Description of Additional Supplementary Files**

File Name: Supplementary Data 1

Description: Nucleotide sequences of primers used for qRT-PCR and ChIP-qPCR
